# Supplementary material for: Ribonucleotide reductase inhibitors suppress SAMHD1 ara‐CTPase activity enhancing cytarabine efficacy
Source: EMBO Mol Med. 2020 Jan 17;12(3):e10419. doi: 10.15252/emmm.201910419 (PMC7059017; doi:10.15252/emmm.201910419)
Supplement: Supplementary file 1 — Appendix [file EMMM-12-e10419-s001.pdf]

Ribonucleotide reductase inhibitors suppress SAMHD1 ara-CTPase activity enhancing cytarabine efficacy

by Rudd, Tsesmetzis, Sanjiv et al.

Table of contents:

Figure S1 | Phenotypic screen for SAMHD1-dependent ara-C sensitisers

Figure S2 | SAMHD1 expression in cell line panel

Figure S3 | Wee1 inhibitor, MK-1775, synergises with ara-C in a SAMHD1-independent manner

Figure S4 | HDAC inhibitors did not sensitise cells to ara-C in a SAMHD1-dependent manner

Figure S5 | Addition of anthracycline did not influence SAMHD1-dependent HU-mediated sensitisation of ara-C

Figure S6 | Ara-C and HU combination treatment in SAMHD1-proficient and deficient THP-1 AML orthotopic xenotransplant mouse model

Figure S7 | Ara-C and HU combination treatment in SAMHD1-proficient and deficient HL60/iva AML orthotopic xenotransplant mouse model

Figure S8 | Ara-C and dF-dC combination treatment in SAMHD1-proficient THP-1 AML orthotopic xenotransplant mouse model.

Figure S9 | SAMHD1 expression in *MLL-AF9* murine AML blasts and sensitisation to ara-C by HU

Figure S10 | Ara-C and RNR inhibitors HU and dF-dC synergise in patient-derived AML blasts in a SAMHD1-dependent manner

Figure S11 | Interrogating the role of reactive oxygen species or phosphorylation status of SAMHD1 at T592 upon ara-C and RNRi synergy

Figure S12 | RNR inhibitors induce nucleotide pool imbalance

Figure S13 | Additional biochemical and thermal shift analyses of SAMHD1

Table S1 | Overview of reported SAMHD1 inhibitors

Table S2 | Characteristics of AML patients

Table S3 | Summary of pre-clinical studies combining irreversible inhibitors of RNR with ara-C

Table of contents (cont.):

Table S4 | Summary of clinical studies combining irreversible inhibitors of ribonucleotide reductase with ara-C

Supplementary References

A

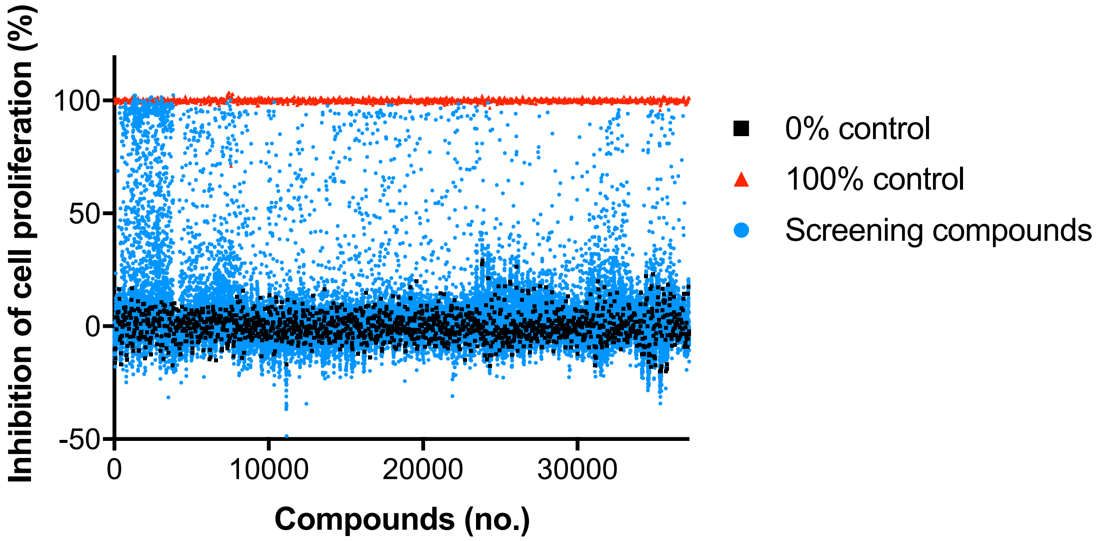
Inhibition of cell proliferation (%)
100
50
0
-50
0
10000
20000
30000
Compounds (no.)
0% control
100% control
Screening compounds

B

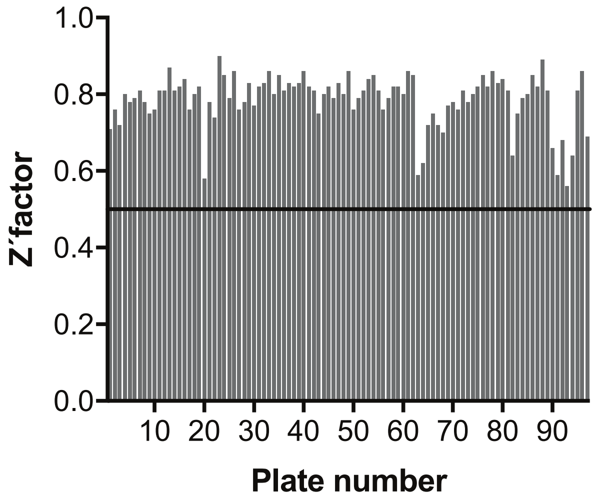
Z´factor
1.0
0.8
0.6
0.4
0.2
0.0
10
20
30
40
50
60
70
80
90
Plate number

C

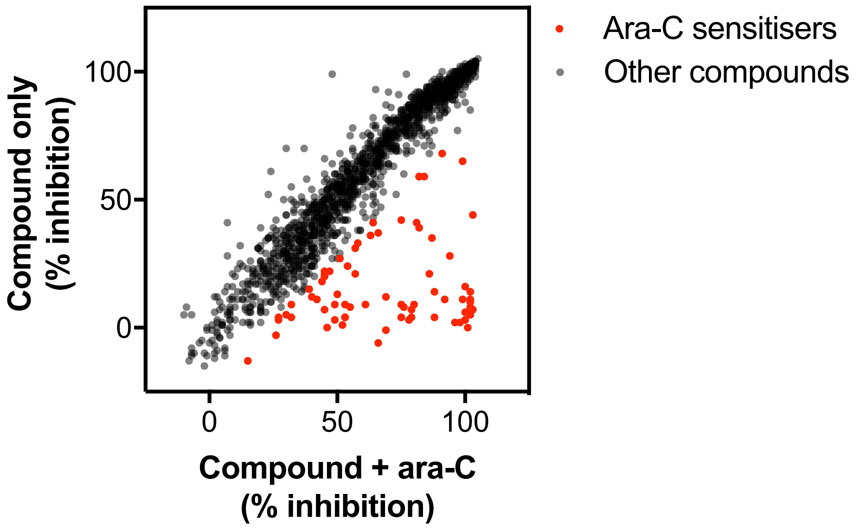
Compound only (% inhibition)
100
50
0
0
50
100
Compound + ara-C (% inhibition)
Ara-C sensitisers
Other compounds

Figure S1 | Phenotypic screen for SAMHD1-dependent ara-C sensitisers

A) SAMHD1+/+ THP-1 cells in the presence of ara-C at an EC_{10} were incubated with a one of 33’467 compounds for 72 h before determining the inhibition of cell proliferation using an ATP-release assay. Percentage of cell proliferation inhibition is indicated by blue circles for compounds, black squares for negative controls, and red triangles for positive controls. For details see **Appendix Supplementary Methods**.

B) Z’ factors for the phenotypic screen per 384-plate. Z’ factors were determined as described before(Zhang et al, 1999). Black line indicates a Z’ factor of 0.5.

C) Approximately 1’600 compounds that inhibited cell proliferation of THP-1 $SAMHD1^{+/+}$ cells in (A) with at least 30% were re-tested the absence or presence of ara-C at an EC_{10}. Grey dots indicate compounds without apparent sensitisation to ara-C whereas red dots indicate compounds that sensitised cells to ara-C.

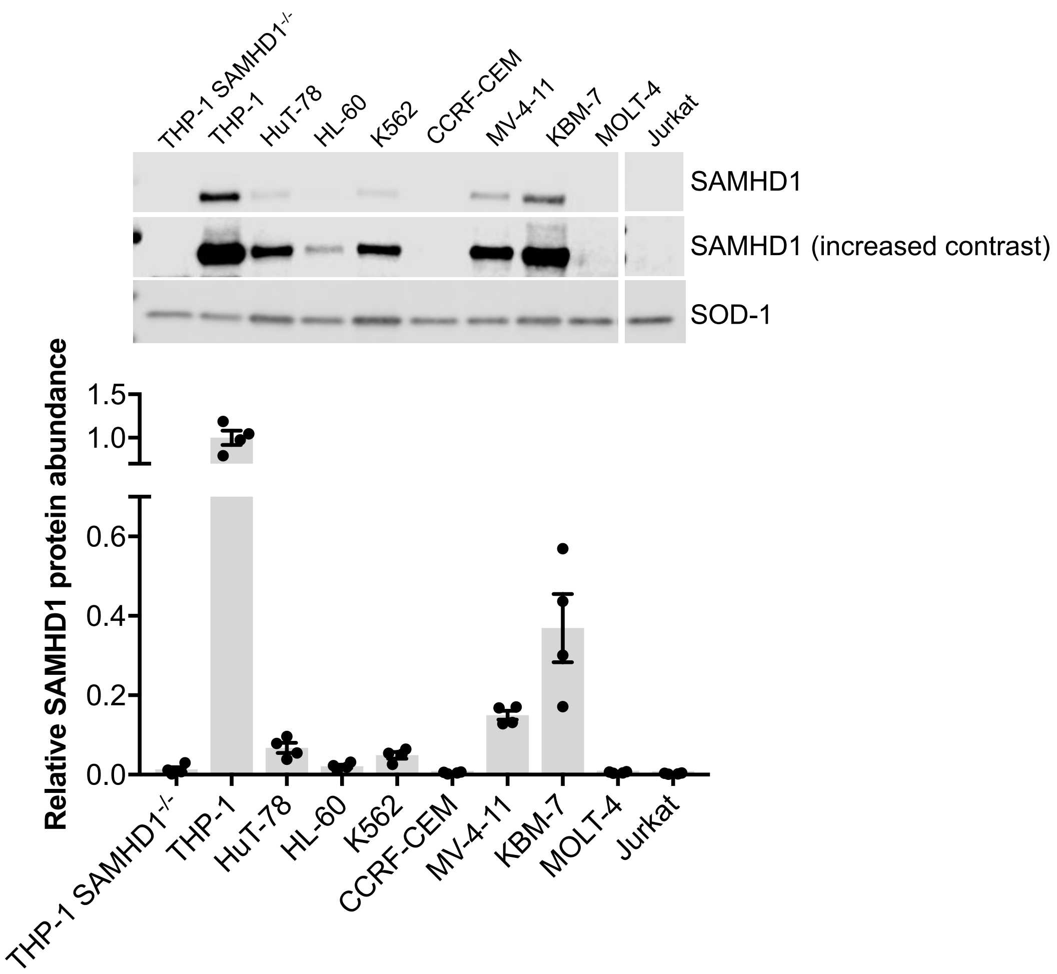
THP-1 SAMHD1-/-
THP-1
HuT-78
HL-60
K562
CCRF-CEM
MV-4-11
KBM-7
MOLT-4
Jurkat
SAMHD1
SAMHD1 (increased contrast)
SOD-1
Relative SAMHD1 protein abundance
1.5
1.0
0.6
0.4
0.2
0.0

Figure S2 | SAMHD1 expression in cell line panel

Western blot analysis of SAMHD1 protein abundance in a panel of haematological cancer cell lines. SAMHD1 protein levels, relative to SOD-1 and normalised to THP-1, from four independent experiments are plotted. Error bars indicate s.e.m.

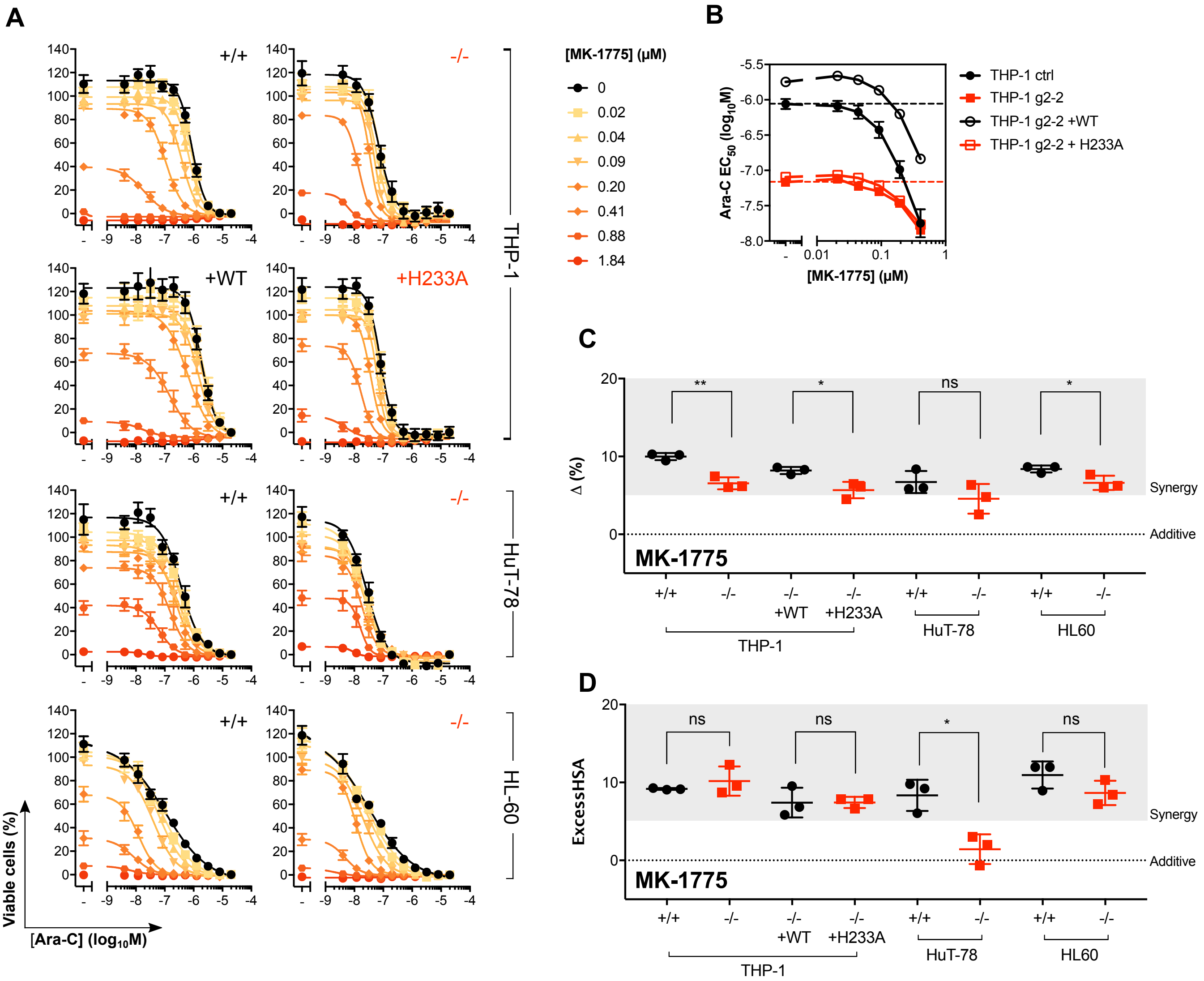
A
+/+
-/-
+WT
+H233A
THP-1
HuT-78
HL-60
Viable cells (%)
[Ara-C] (log10M)
[MK-1775] (µM)
0
0.02
0.04
0.09
0.20
0.41
0.88
1.84
B
Ara-C EC50 (log10M)
[MK-1775] (µM)
THP-1 ctrl
THP-1 g2-2
THP-1 g2-2 +WT
THP-1 g2-2 + H233A
C
Δ (%)
MK-1775
Synergy
Additive
**
*
ns
*
+/+
-/-
-/- +WT
-/- +H233A
THP-1
HuT-78
HL60
D
ExcessHSA
MK-1775
ns
ns
*
ns
Synergy
Additive

Figure S3 | Wee1 inhibitor, MK-1775, synergises with ara-C in a SAMHD1-independent manner

A) Proliferation inhibition analysis of ara-C and MK-1775 combination treatment in SAMHD1-proficient ($^{+/+}$), deficient ($^{–/–}$) and rescue (WT, wild-type; H233A, catalytic-dead) cell line pairs. Error bars indicate s.e.m. of 3 independent experiments each performed in duplicate.

B) Half-maximal effective concentration (EC_{50}) values for ara-C plotted as a function of MK-1775 concentration in SAMHD1-proficient ($^{+/+}$), deficient ($^{–/–}$) and rescue (WT, wild-type; H233A, catalytic-dead) THP-1 cell line pairs. Ara-C EC_{50} values for SAMHD1$^{+/+}$ and SAMHD1$^{–/–}$ THP-1 cells in the absence of MK-1775 is indicated by the black and red dotted line, respectively. Error bars indicate s.e.m. of three independent experiments each performed in duplicate.

C) Drug synergy plots for ara-C and MK-1775 in the SAMHD1-proficient ($^{+/+}$), deficient ($^{–/–}$) and rescue (WT, wild-type; H233A, catalytic-dead) cell line pairs. Zero, >0, or <0 corresponds to additive, synergy, or antagonism, respectively, whilst >5 indicates strong synergy. Each data point indicates an average delta score from a single dose-response matrix experiment performed in duplicate. The horizontal line and the error bars indicate the mean and s.d., respectively, statistical significance was determined using a two-tailed unpaired *t*-test: ns, not significant, $P \geq 0.05$; *, $P < 0.05$; **, $P < 0.01$.

D) Drug synergy plots for ara-C and MK-1775 in the SAMHD1-proficient ($^{+/+}$), deficient ($^{–/–}$) and rescue (WT, wild-type; H233A, catalytic-dead) cell line pairs. Zero, >0, or <0 corresponds to additive, synergy, or antagonism, respectively, >5 indicates strong synergy. Each data point indicates an average excess HSA synergy score from a single dose-response matrix experiment performed in duplicate. The horizontal line and the error bars indicate the mean and s.d., respectively, statistical significance was determined using a two-tailed unpaired *t*-test: ns, not significant, $P \geq 0.05$; *, $P < 0.05$.

Data information: Detail of statistical testing in (C): For THP-1 $^{+/+}$ vs $^{–/–}$, $n = 3$, $P = 0.0027$, $t = 6.634$, df = 4; THP-1 WT vs H233A, $n = 3$, $P = 0.0194$, $t = 3.782$, df = 4; HuT-78 $^{+/+}$ vs $^{–/–}$, $n = 3$, $P = 0.1918$, $t = 1.569$, df = 4; HL-60 $^{+/+}$ vs $^{–/–}$, $n = 3$, $P = 0.0399$, $t = 3.002$, df = 4. Details of statistical testing in (D): For THP-1 $^{+/+}$ vs $^{–/–}$, $n = 3$, $P = 0.4020$, $t = 0.9366$, df = 4; THP-1 WT vs H233A, $n = 3$, $P = 0.9851$, $t = 0.01989$, df = 4; HuT-78 $^{+/+}$ vs $^{–/–}$, $n = 3$, $P = 0.0123$, $t = 4.332$, df = 4; HL-60 $^{+/+}$ vs $^{–/–}$, $n = 3$, $P = 0.1654$, $t = 1.695$, df = 4.

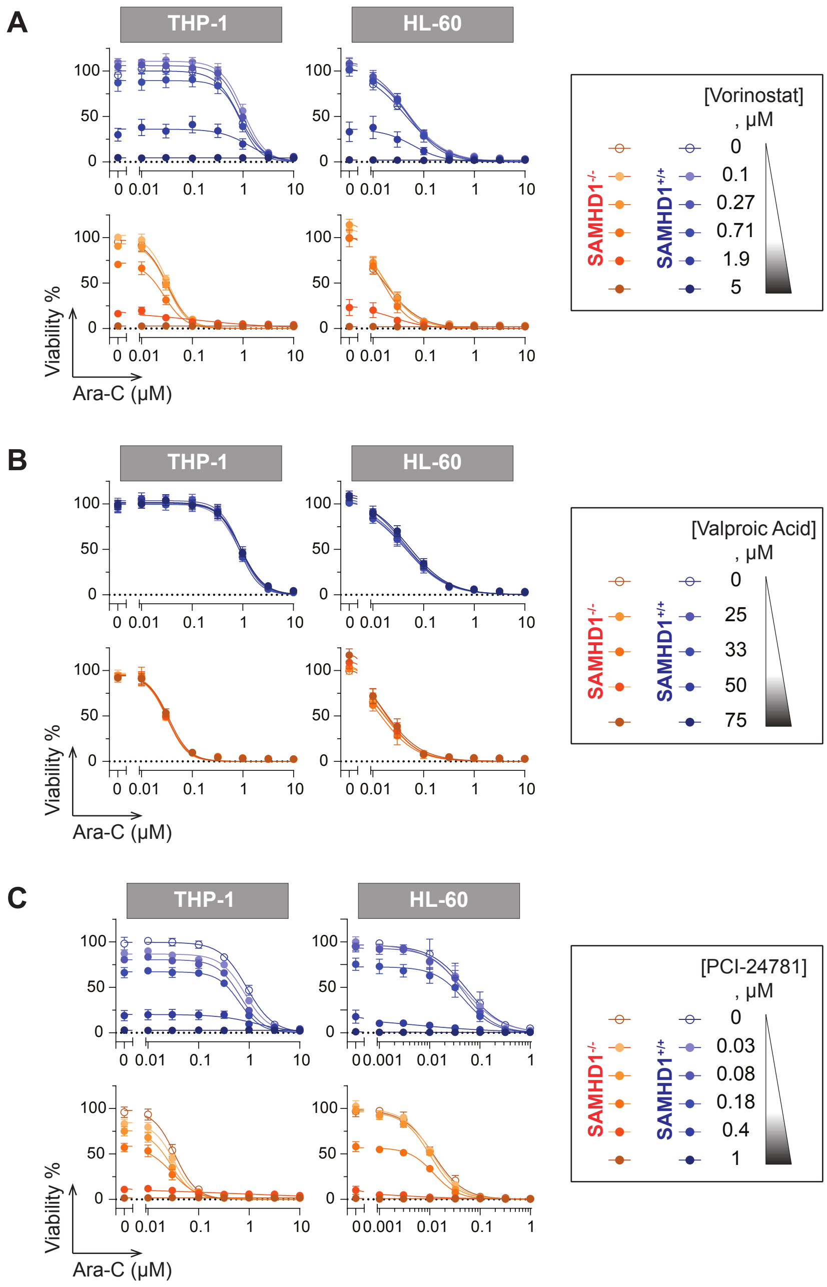
A
THP-1
HL-60
Viability %
Ara-C (µM)
[Vorinostat], µM
SAMHD1-/-
SAMHD1+/+
0
0.1
0.27
0.71
1.9
5
B
THP-1
HL-60
Viability %
Ara-C (µM)
[Valproic Acid], µM
SAMHD1-/-
SAMHD1+/+
0
25
33
50
75
C
THP-1
HL-60
Viability %
Ara-C (µM)
[PCI-24781], µM
SAMHD1-/-
SAMHD1+/+
0
0.03
0.08
0.18
0.4
1

Figure S4 | HDAC inhibitors did not sensitise cells to ara-C in a SAMHD1-dependent manner

SAMHD1-proficient ($^{+/+}$) or -deficient ($^{-/-}$) THP-1 or HL-60 cells were treated with a dose-response matrix composed of ara-C and an HDAC inhibitor, either vorinostat (A), valproic acid (B) or PCI-24781 (C). Four days post-treatment, cell viabilities were assayed using resazurin reduction viability assay.

Data information: Cell viability percentages (%) were calculated by normalizing to DMSO-treated controls. Mean viability % ± s.e.m. of n = 2-3 were shown with viability curves (non-linear regression, GraphPad Prism).

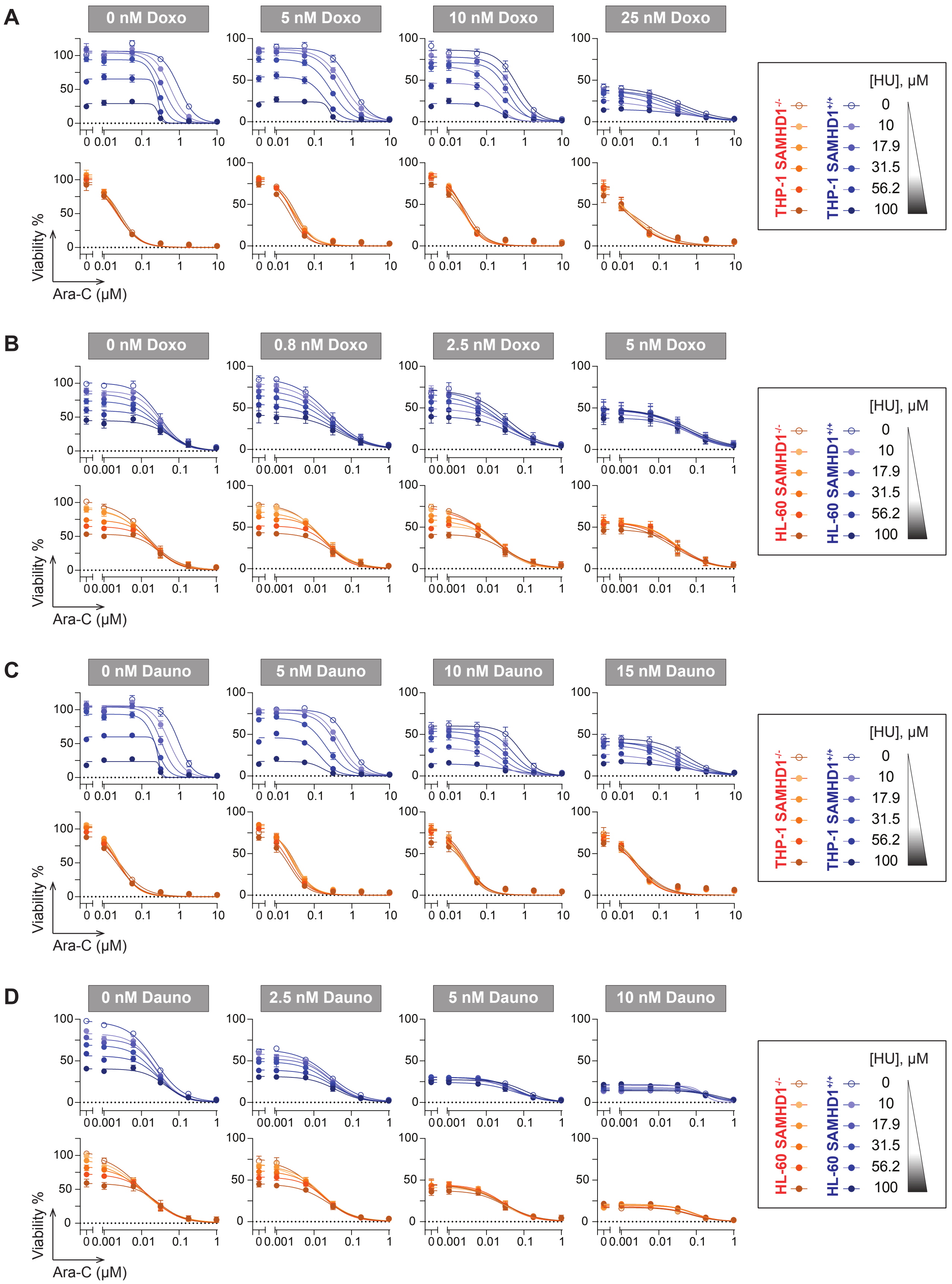
A
0 nM Doxo
5 nM Doxo
10 nM Doxo
25 nM Doxo
[HU], µM
THP-1 SAMHD1-/-
THP-1 SAMHD1+/+
0
10
17.9
31.5
56.2
100
Viability %
Ara-C (µM)
B
0 nM Doxo
0.8 nM Doxo
2.5 nM Doxo
5 nM Doxo
HL-60 SAMHD1-/-
HL-60 SAMHD1+/+
C
0 nM Dauno
5 nM Dauno
10 nM Dauno
15 nM Dauno
D
0 nM Dauno
2.5 nM Dauno
5 nM Dauno
10 nM Dauno

Figure S5 | Addition of anthracycline did not influence SAMHD1-dependent HU-mediated sensitisation of ara-C

SAMHD1-proficient ($^{+/+}$) or -deficient ($^{-/-}$) THP-1 (A, C) or HL-60 (B, D) cells were treated with a dose-response matrix of HU and ara-C, in the absence or presence of increasing concentrations of doxorubicin (Doxo; A, B) or daunorubicin (Dauno; C, D) for four days before cell viabilities were determined using resazurin reduction viability assay.

Data information: Cell viability percentages (%) were calculated by normalizing to DMSO-treated controls. Mean viability % ± s.e.m. of $n \geq 3$ were shown with viability curves (non-linear regression, GraphPad Prism).

A

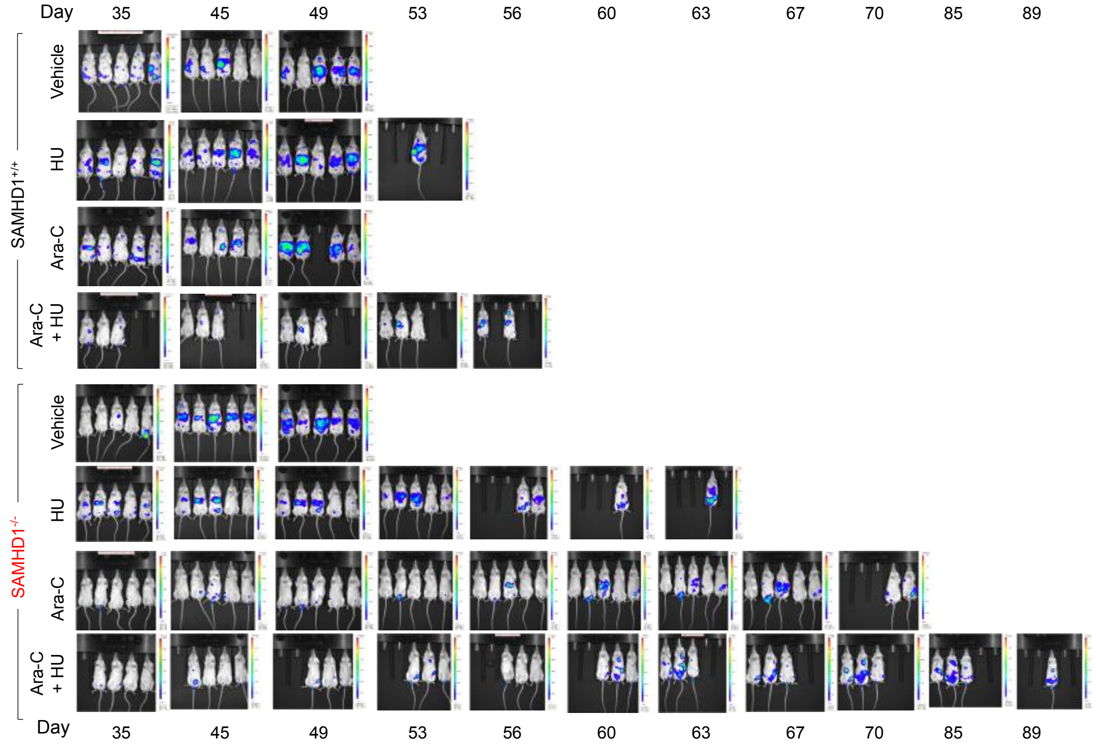

B

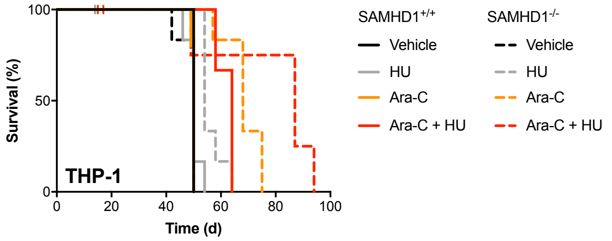

C

Log-rank (Mantel-Cox) test		+/+				-/-			
		Vehicle	Ara-C	HU	Ara-C + HU	Vehicle	Ara-C	HU	Ara-C + HU
+/+	Vehicle								
	Ara-C	0.3173							
	HU	0.9486							
	Ara-C + HU	0.0082	0.0141	0.0079					
-/-	Vehicle	0.3613			0.0095				
	Ara-C		0.0018		0.054	0.0012			
	HU			0.0056	0.109	0.0012			
	Ara-C + HU				0.1691	0.0838	0.0737	0.0596	

ns
*
**

D

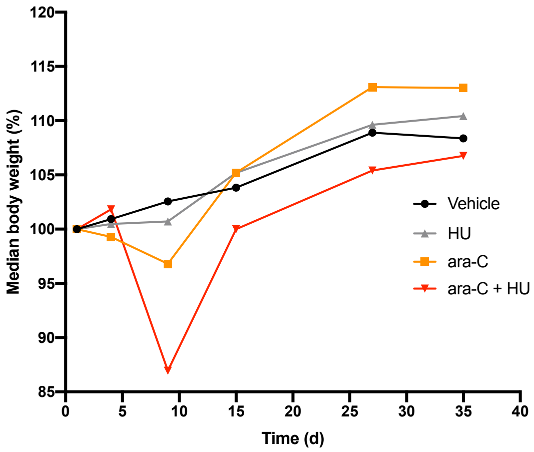

E

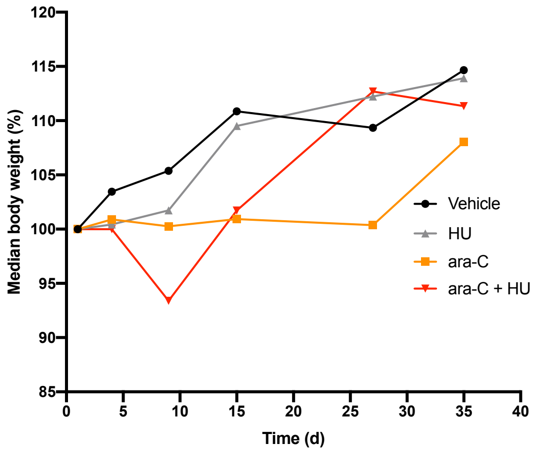

Figure S6 | Ara-C and HU combination treatment in SAMHD1-proficient and deficient THP-1 AML orthotopic xenotransplant mouse model

A) Representative images of NOD/SCID mice (*n* = 6 per treatment group) injected i.v. with luciferase-expressing SAMHD1-proficient ($^{+/+}$) or deficient ($^{-/-}$) THP-1 cell clones (day 0) and treated with ara-C and/or HU i.p. as indicated (day 6). On the indicated days, mice were injected with D-luciferin i.p. to monitor disease progression.

B) Kaplan-Meier analysis, tick marks indicate censored animals.

C) *P* value matrix comparing indicated treatment groups using Log-rank Mantel Cox test: ns, not significant, $P \geq 0.05$, white; *, $P < 0.05$, blue; **, $P < 0.01$, red.

D) Median body weight of animals per treatment group bearing THP-1 SAMHD1$^{+/+}$ xenotransplants during the experiment (day 1 until day 35).

E) Median body weight of animals per treatment group bearing THP-1 SAMHD1$^{-/-}$ xenotransplants during the experiment (day 1 until day 35).

Data information: Details of statistical testing in (C): for $^{+/+}$ vehicle vs $^{+/+}$ HU, *n* = 5 and 6, respectively, $P = 0.9486$, $\chi^2 = 0.004149$, df = 1; $^{+/+}$ vehicle vs $^{+/+}$ ara-C, *n* = 5 and 6, respectively, $P = 0.3173$, $\chi^2 = 1$, df = 1; $^{+/+}$ vehicle vs $^{+/+}$ ara-C + HU, *n* = 5 and 3, respectively, $P = 0.0082$, $\chi^2 = 7$, df = 1; $^{+/+}$ HU vs $^{+/+}$ ara-C + HU, *n* = 6 and 3, respectively, $P = 0.0079$, $\chi^2 = 7.059$, df = 1; $^{+/+}$ ara-C vs $^{+/+}$ ara-C + HU, *n* = 5 and 3, respectively, $P = 0.0141$, $\chi^2 = 6.028$, df = 1. For $^{-/-}$ vehicle vs $^{-/-}$ HU, *n* = 6, $P = 0.0012$, $\chi^2 = 10.48$, df = 1, $^{-/-}$ vehicle vs $^{-/-}$ ara-C, *n* = 6, $P = 0.0012$, $\chi^2 = 10.48$, df = 1, $^{-/-}$ vehicle vs $^{-/-}$ ara-C + HU, *n* = 6 and 4, respectively, $P = 0.0838$, $\chi^2 = 2.989$, df = 1; $^{-/-}$ HU vs $^{-/-}$ ara-C + HU, *n* = 6 and 4, respectively, $P = 0.0596$, $\chi^2 = 3.549$, df = 1; $^{-/-}$ ara-C vs $^{-/-}$ ara-C + HU, *n* = 6 and 4, respectively, $P = 0.0737$, $\chi^2 = 3.199$, df = 1. For $^{+/+}$ vehicle vs $^{-/-}$ vehicle, *n* = 5 and 6, respectively, $P = 0.3613$, $\chi^2 = 0.8333$, df = 1; $^{+/+}$ ara-C vs $^{-/-}$ ara-C, *n* = 5 and 6, respectively, $P = 0.0018$, $\chi^2 = 9.771$, df = 1; $^{+/+}$ HU vs $^{-/-}$ HU, *n* = 6, $P = 0.0056$, $\chi^2 = 7.669$, df = 1; $^{+/+}$ ara-C + HU vs $^{-/-}$ ara-C + HU, *n* = 3 and 4, respectively, $P = 0.1691$, $\chi^2 = 1.891$, df = 1; $^{+/+}$ ara-C + HU vs $^{-/-}$ vehicle, *n* = 3 and 5, respectively, $P = 0.0095$, $\chi^2 = 6.732$, df = 1; $^{+/+}$ ara-C + HU vs $^{-/-}$ ara-C, *n* = 3 and 6, respectively, $P = 0.0540$, $\chi^2 = 3.714$, df = 1; $^{+/+}$ ara-C + HU vs $^{-/-}$ HU, *n* = 3 and 6, respectively, $P = 0.1090$, $\chi^2 = 2.568$, df = 1.

A

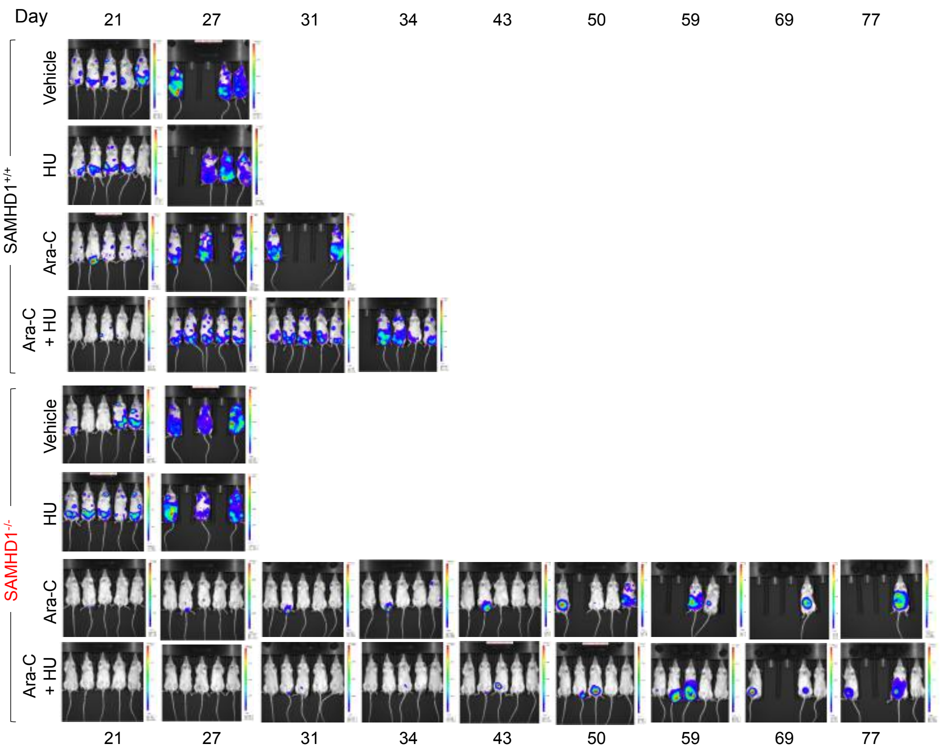

B

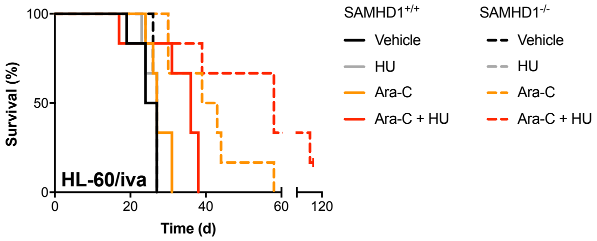

C

Log-rank (Mantel-Cox) test		+/+ Vehicle	+/+ Ara-C	+/+ HU	+/+ Ara-C + HU	-/- Vehicle	-/- Ara-C	-/- HU	-/- Ara-C + HU
+/+	Vehicle								
+/+	Ara-C	0.1845							
+/+	HU	0.5999							
+/+	Ara-C + HU	0.0186	0.0316	0.022					
-/-	Vehicle	0.375			0.0217				
-/-	Ara-C		0.015		0.0715	0.0012			
-/-	HU			0.8801	0.022	0.8821			
-/-	Ara-C + HU				0.0123	0.0012	0.0893	0.0012	

ns
*
**

D

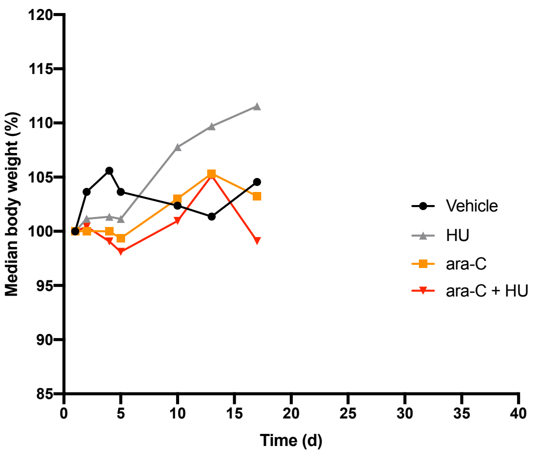

E

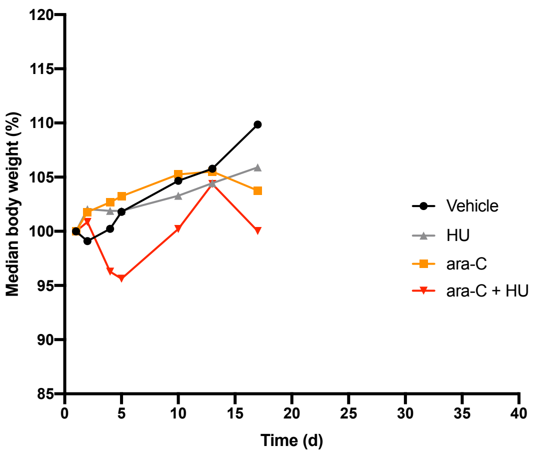

Figure S7 | Ara-C and HU combination treatment in SAMHD1-proficient and deficient HL60/iva AML orthotopic xenotransplant mouse model

A) Representative images of NOD/SCID mice (*n* = 6 per treatment group) injected i.v. with luciferase-expressing SAMHD1-proficient ($^{+/+}$) or deficient ($^{-/-}$) HL-60/iva cell clones (day 0) and treated with ara-C and/or HU as indicated (day 6). On the indicated days, mice were injected with D-luciferin to monitor disease progression.

B) Kaplan-Meier analysis, tick marks indicate censored animals.

C) *P* value matrix comparing indicated treatment groups using Log-rank Mantel Cox test: ns, not significant, $P \geq 0.05$, white; *, $P < 0.05$, blue; **, $P < 0.01$, red.

D) Median body weight of animals per treatment group bearing HL60/iva SAMHD1$^{+/+}$ xenotransplants during the experiment (day 1 until day 17).

E) Median body weight of animals per treatment group bearing HL60/iva SAMHD1$^{-/-}$ xenotransplants during the experiment (day 1 until day 17).

Data information: Details of statistical testing in (C): for $^{+/+}$ vehicle vs $^{+/+}$ HU, $n = 6$, $P = 0.5999$, $\chi^2 = 0.2752$, df = 1; $^{+/+}$ vehicle vs $^{+/+}$ ara-C, n =6, $P = 0.1845$, $\chi^2 = 1.761$, df = 1; $^{+/+}$ vehicle vs $^{+/+}$ ara-C + HU, $n = 6$, $P = 0.0186$, $\chi^2 = 5.542$, df = 1; $^{+/+}$ HU vs $^{+/+}$ ara-C + HU, $n = 6$, $P = 0.0220$, $\chi^2 = 5.248$, df = 1, $^{+/+}$ ara-C vs $^{+/+}$ ara-C + HU, $n = 6$, $P = 0.0316$, $\chi^2 = 4.619$, df = 1. For $^{-/-}$ vehicle vs $^{-/-}$ HU, $n = 6$, $P = 0.8821$, $\chi^2 = 0.022$, df = 1, $^{-/-}$ vehicle vs $^{-/-}$ ara-C, $n = 6$, $P = 0.0012$, $\chi^2 = 10.56$, df = 1, $^{-/-}$ vehicle vs $^{-/-}$ ara-C + HU, n = 6 and 5, respectively, $P = 0.0012$, $\chi^2 = 10.56$, df = 1; $^{-/-}$ HU vs $^{-/-}$ ara-C + HU, n = 6 and 5, respectively, $P = 0.0012$, $\chi^2 = 10.43$, df = 1; $^{-/-}$ ara-C vs $^{-/-}$ ara-C + HU, n = 6 and 5, respectively, $P = 0.0893$, $\chi^2 = 2.886$, df = 1. For $^{+/+}$ vehicle vs $^{-/-}$ vehicle, $n = 6$, $P = 0.3750$, $\chi^2 = 0.7872$, df = 1; $^{+/+}$ ara-C vs $^{-/-}$ ara-C, $n = 6$, $P = 0.0150$, $\chi^2 = 5.918$, df = 1; $^{+/+}$ HU vs $^{-/-}$ HU, $n = 6$, $P = 0.8801$, $\chi^2 = 0.02274$, df = 1; $^{+/+}$ ara-C + HU vs $^{-/-}$ ara-C + HU, n = 6 and 5, respectively, $P = 0.0123$, $\chi^2 = 6.261$, df = 1; $^{+/+}$ ara-C + HU vs $^{-/-}$ vehicle, $n = 6$, $P = 0.0217$, $\chi^2 = 5.271$, df = 1; $^{+/+}$ ara-C + HU vs $^{-/-}$ ara-C, $n = 6$, $P = 0.0715$, $\chi^2 = 3.248$, df = 1; $^{+/+}$ ara-C + HU vs $^{-/-}$ HU, $n = 6$, $P = 0.0220$, $\chi^2 = 5.248$, df = 1.

A

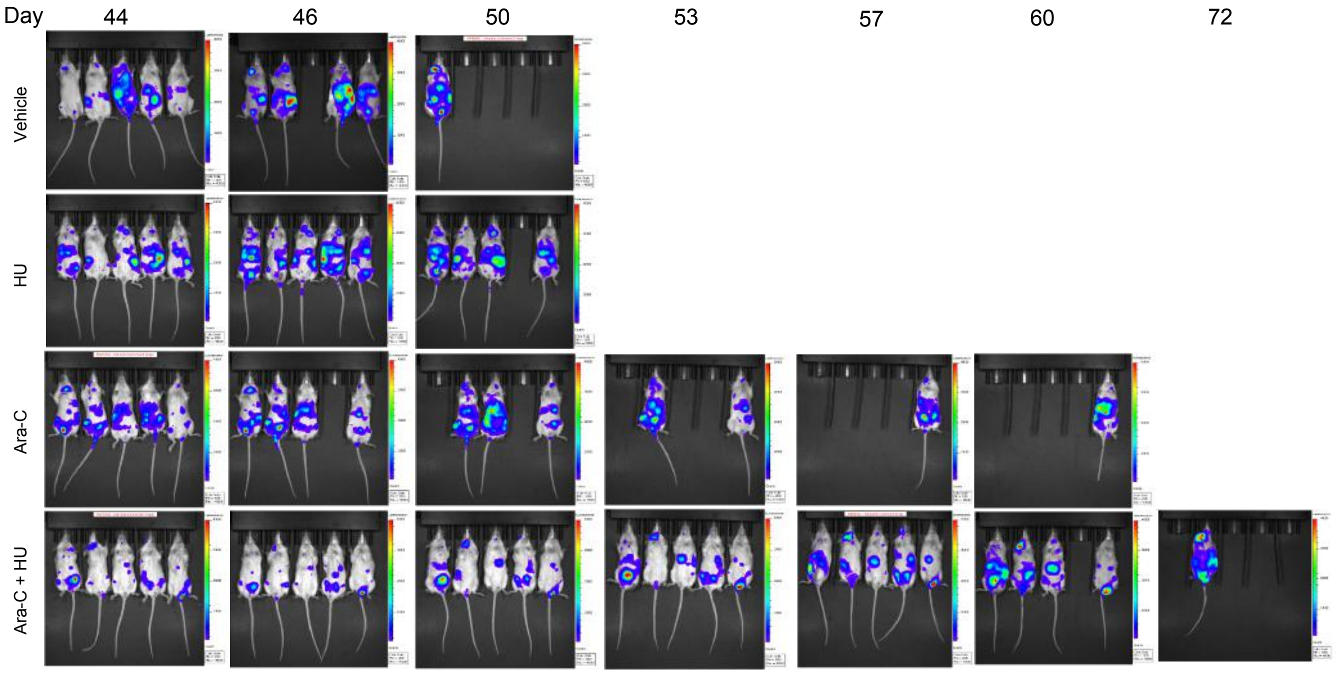
Day
44
46
50
53
57
60
72
Vehicle
HU
Ara-C
Ara-C + HU

B

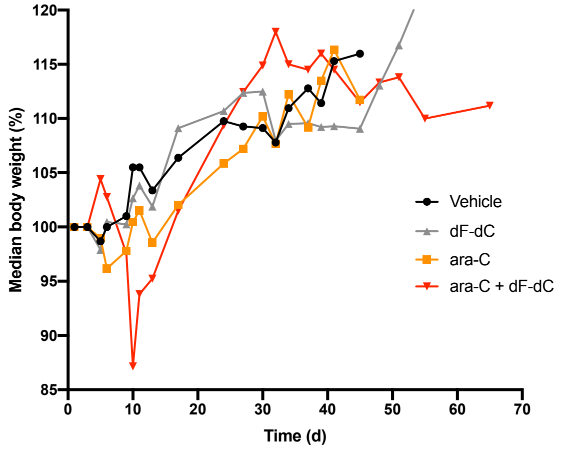
Median body weight (%)
120
115
110
105
100
95
90
85
0
10
20
30
40
50
60
70
Time (d)
Vehicle
dF-dC
ara-C
ara-C + dF-dC

Figure S8 | Ara-C and dF-dC combination treatment in SAMHD1-proficient THP-1 AML orthotopic xenotransplant mouse model

A) Representative images of NOD/SCID mice (*n* = 7 per treatment group) injected i.v. with luciferase-expressing SAMHD1-proficient THP-1 cell clone (day 0) and treated with ara-C and/or dF-dC as indicated (day 6). On the indicated days, mice were injected with D-luciferin to monitor disease progression.

B) Median body weight of animals per treatment group during the experiment (day 1 until day 65).

A

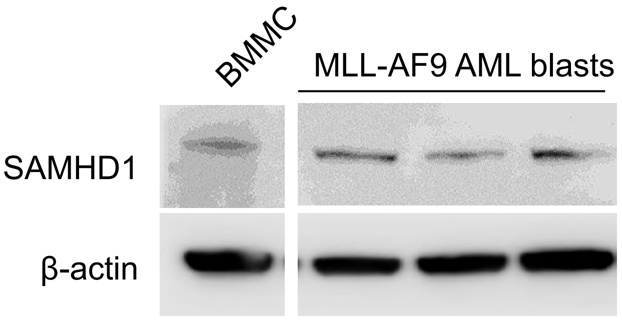

BMMC
MLL-AF9 AML blasts
SAMHD1
β-actin

B

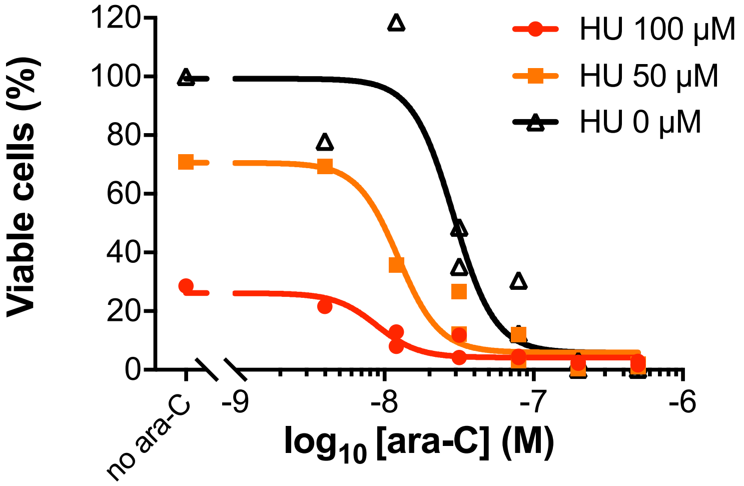

Viable cells (%)
120
100
80
60
40
20
0
HU 100 µM
HU 50 µM
HU 0 µM
no ara-C
-9
-8
-7
-6
log10 [ara-C] (M)

Figure S9 | SAMHD1 expression in *MLL-AF9* murine AML blasts and sensitisation to ara-C by HU

A) Immunoblot of murine bone mononuclear cells (BMMCs, left lane) and *MLL-AF9* murine AML blast (right lanesw) lysates stained for SAMHD1 and β-actin using a polycloncal rabbit anti-SAMHD1 antibody (Proteintech, 12586-1-AP) and a monoclonal mouse anti-β-actin IgG1 coupled to horse-radish peroxidase (Santa Cruz Biotechnology, sc-47778 HRP).

B) *MLL-AF9* murine AML blast were treated with increasing concentrations of ara-C for 72 h in the presence of HU at the indicated concentrations prior to determining cell viability using an ATP-release assay. Calculated EC_{50} values are 29.9 nM, 12.4 nM, and 8.9 nM for 0 μM, 50 μM, and 100 μM of HU, respectively, Curve fitting was performed using non-linear regression in Prism 7. Extra sum-of-squares F test showed was performed: $F = 25.96$; DFn = 2; DFd = 55; $P < 0.0001$.

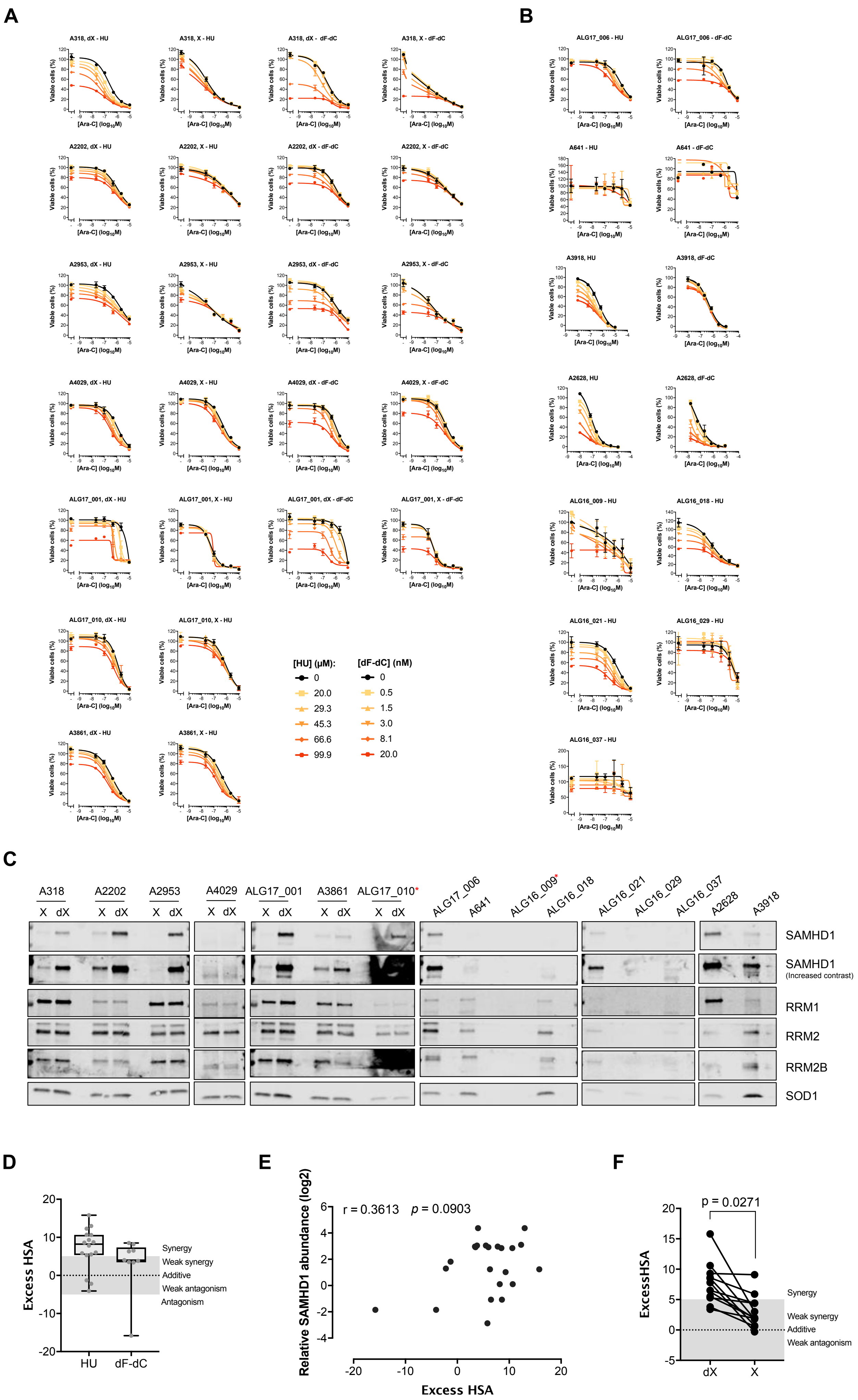
A
B
A318, dX - HU
A318, X - HU
A318, dX - dF-dC
A318, X - dF-dC
A2202, dX - HU
A2202, X - HU
A2202, dX - dF-dC
A2202, X - dF-dC
A2953, dX - HU
A2953, X - HU
A2953, dX - dF-dC
A2953, X - dF-dC
A4029, dX - HU
A4029, X - HU
A4029, dX - dF-dC
A4029, X - dF-dC
ALG17_001, dX - HU
ALG17_001, X - HU
ALG17_001, dX - dF-dC
ALG17_001, X - dF-dC
ALG17_010, dX - HU
ALG17_010, X - HU
A3861, dX - HU
A3861, X - HU
ALG17_006 - HU
ALG17_006 - dF-dC
A641 - HU
A641 - dF-dC
A3918, HU
A3918, dF-dC
A2628, HU
A2628, dF-dC
ALG16_009 - HU
ALG16_018 - HU
ALG16_021 - HU
ALG16_029 - HU
ALG16_037 - HU
Viable cells (%)
[Ara-C] (log10M)
[HU] (µM):
0
20.0
29.3
45.3
66.6
99.9
[dF-dC] (nM)
0
0.5
1.5
3.0
8.1
20.0
C
A318
A2202
A2953
A4029
ALG17_001
A3861
ALG17_010*
X dX
ALG17_006
A641
ALG16_009*
ALG16_018
ALG16_021
ALG16_029
ALG16_037
A2628
A3918
SAMHD1
SAMHD1
(Increased contrast)
RRM1
RRM2
RRM2B
SOD1
D
Excess HSA
Synergy
Weak synergy
Additive
Weak antagonism
Antagonism
HU
dF-dC
E
r = 0.3613 p = 0.0903
Relative SAMHD1 abundance (log2)
Excess HSA
F
p = 0.0271
ExcessHSA
Synergy
Weak synergy
Additive
Weak antagonism
dX
X

Figure S10 | Ara-C and RNR inhibitors HU and dF-dC synergise in patient-derived AML blasts in a SAMHD1-dependent manner

A) Proliferation inhibition analysis of ara-C and RNRi (HU or dF-dC) combination treatment in patient-derived AML blasts pre-treated with control (dX) or Vpx-containing (X) VLPs. Error bars indicate s.d. of a single experiment performed in triplicate. Panels "A2953, dX – HU" and "A2953, X – HU" are identical to the panels shown in Fig 3D. Panels "ALG17_001, dX – dF-dC" and "ALG17_001, X – dF-dC" are identical to the panels shown in Fig 3F. Clinicopathological features can be found in Table S2.

B) Proliferation inhibition analysis of ara-C and RNRi (HU or dF-dC) combination treatment in patient-derived AML blasts. Error bars indicate s.d. of a single experiment performed in triplicate, with the exception of sample A641 for ara-C vs dF-dC, which is data obtained in singlet. Clinicopathological features can be found in Table S2.

C) Immunoblot analysis of patient-derived AML blasts pre-treated or not with control (dX) or Vpx-containing (X) VLPs. The red asterisk indicates samples omitted from quantification.

D) Drug synergy plots for ara-C and HU or dF-dC in primary patient-derived AML blasts. Zero, >0, or <0 corresponds to additive, synergy, or antagonism, respectively, whilst >5 indicates strong synergy and <5 indicates strong antagonism. Each data point indicates an average excess HSA score from a single patient sample subjected to a dose-response matrix experiment performed in triplicate, $n = 16$ for HU and $n = 9$ for dF-dC. Median, upper and lower quartiles, and range of excess HSA scores are indicated by box-and-whisker plots. For proliferation inhibition curves for each sample see (A) and (B), and for patient characteristics see **Appendix Table S2**.

E) Pearson correlation of relative SAMHD1 protein abundance and excess HSA synergy scores for ara-C and HU or dF-dC in primary patient-derived AML blasts ($n = 23$). For Western blot analysis of SAMHD1 protein abundance see (C).

F) Paired drug synergy plot for ara-C and RNRi (HU, $n = 7$; dF-dC, $n = 5$) in primary patient-derived AML blasts pre-treated with control (dX) or Vpx-containing (X) VLPs. Each data point indicates an average excess HSA score from a single patient sample subjected to a dose-

response matrix experiment performed in triplicate. Statistical significance determined using two-way ANOVA, $n = 12$, $F = 11.6$, $dF = 1$.

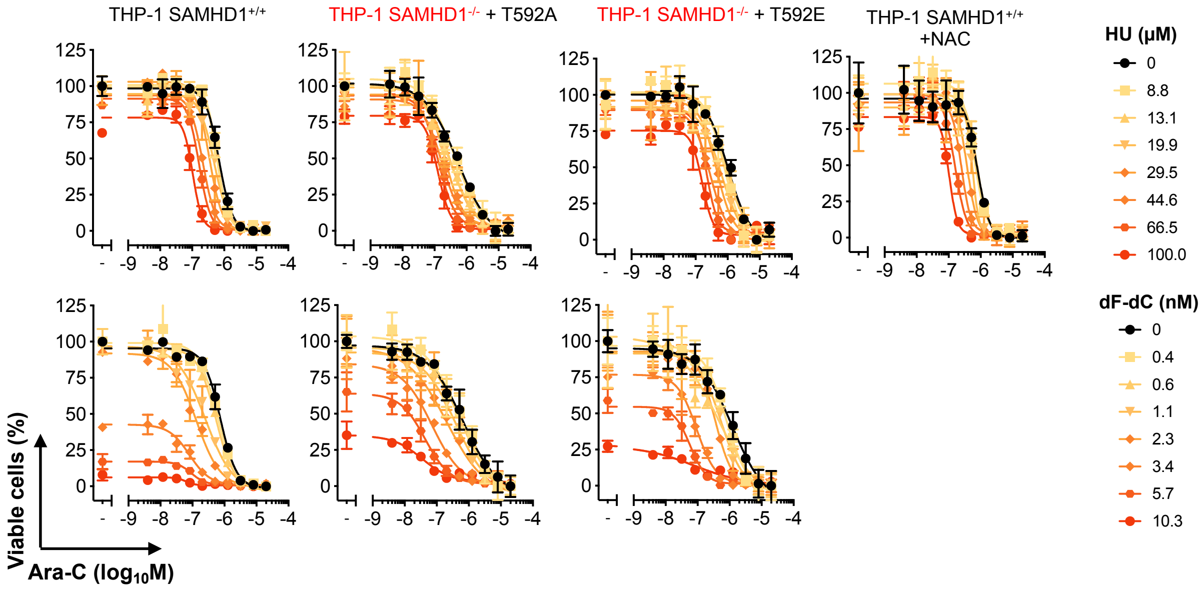
THP-1 SAMHD1+/+
THP-1 SAMHD1-/- + T592A
THP-1 SAMHD1-/- + T592E
THP-1 SAMHD1+/+ +NAC
HU (µM)
0
8.8
13.1
19.9
29.5
44.6
66.5
100.0
dF-dC (nM)
0
0.4
0.6
1.1
2.3
3.4
5.7
10.3
Viable cells (%)
Ara-C (log10M)

Figure S11 | Interrogating the role of reactive oxygen species or phosphorylation status of SAMHD1 at T592 upon ara-C and RNRi synergy

Proliferation inhibition analysis of ara-C and RNRi (HU or dF-dC) in SAMHD1-proficient ($^{+/+}$) or -deficient ($^{-/-}$) THP-1 cells, the latter with rescue expression of phosphomimic (T592E) or phosphorylation-null (T592A) SAMHD1 mutant, or pre-treatment with ROS scavenger *N*-acetylcysteine (NAC, 5 mM for 4 hrs), as indicated. Representative of 2 experiments shown, error bars indicate s.d. of experimental triplicates.

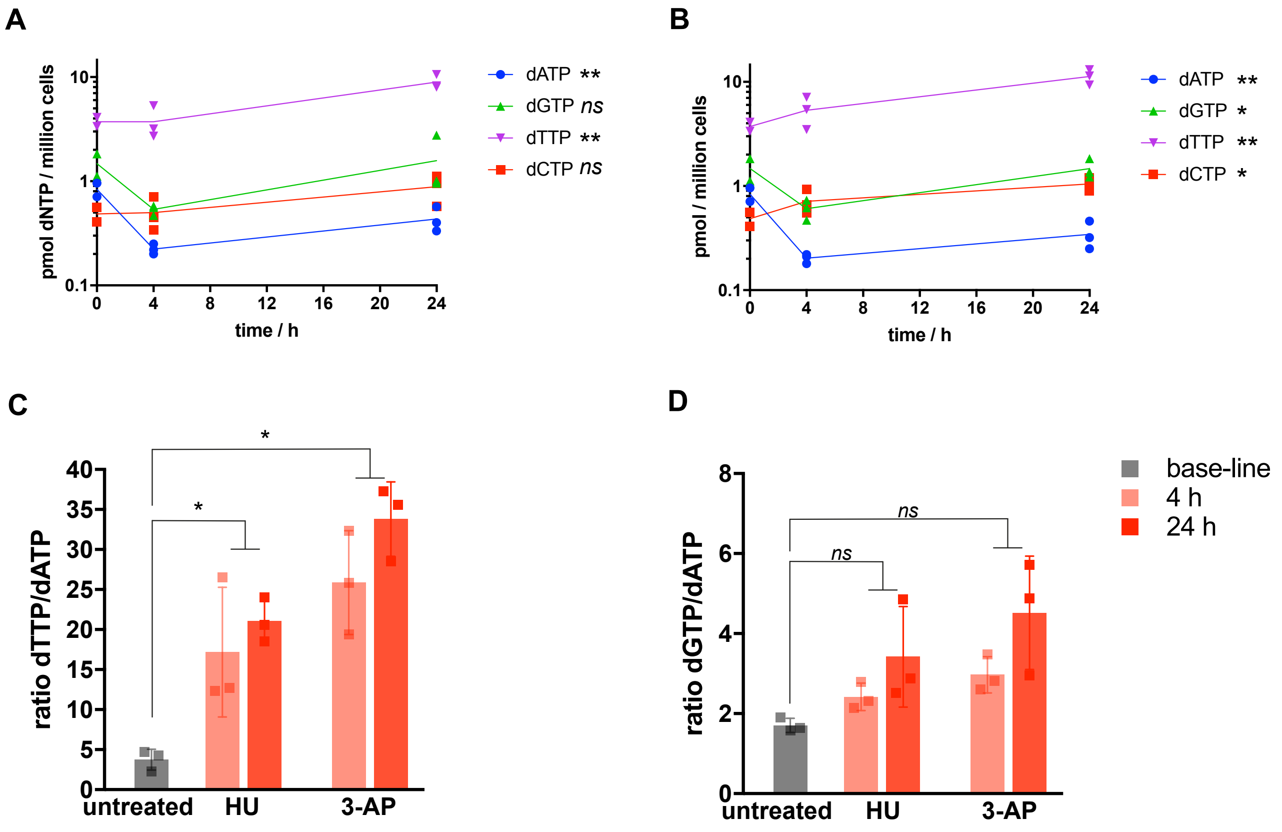
A
pmol dNTP / million cells
time / h
dATP **
dGTP ns
dTTP **
dCTP ns
B
pmol / million cells
time / h
dATP **
dGTP *
dTTP **
dCTP *
C
ratio dTTP/dATP
untreated
HU
3-AP
D
ratio dGTP/dATP
untreated
HU
3-AP
ns
base-line
4 h
24 h

Figure S12 | RNR inhibitors induce nucleotide pool imbalance

A-B) THP-1 SAMHD1$^{+/+}$ cells were treated for 0 h, 4 h or 24 h with either 50 µM HU (A) or 2.5 nM 3-AP (B) prior to determination of intracellular amounts of dNTPs using a primer-extension assay. Individual dots represent single measurements of dATP (blue), dGTP (green), dTTP (purple), or dCTP (red) for a total of three independent experiments. Statistical analyses were performed using one-way ANOVA for each dNTP. HU: dATP: F=18; R^2=0.8786; *P*=0.0051. dGTP: F=1.985; R^2=0.4426; *P*=0.2320. dTTP: F=15.67; R^2=0.8624; *P*=0.0070. dCTP: F=2.936; R^2=0.5423; *P*=0.1417. 3-AP: dATP: F=22.85; R^2=0.9014; *P*=0.0031. dGTP: F=7.159; R^2=0.7412; *P*=0.0341. dTTP: F=14.93; R^2=0.8566; *P*=0.0078. dCTP: F=7.544; R^2=0.7511; *P*=0.0309.

C-D) Ratios of dGTP-to-dATP (C) and dTTP-to-dATP (D) were calculated and normalised to untreated cells. Three independent experiments were performed. Statistical analyses were done using unpaired two-tailed *t*-tests. c, HU vs. untreated: t = 2.422; df = 2; *P* = 0.1364. 3-AP vs. untreated: t = 2.629; df = 2; *P* = 0.1193. d, HU vs. untreated: t = 7.928; df = 2; *P* = 0.0155. 3-AP vs. untreated: t = 6.57; df = 2; *P* = 0.0224.

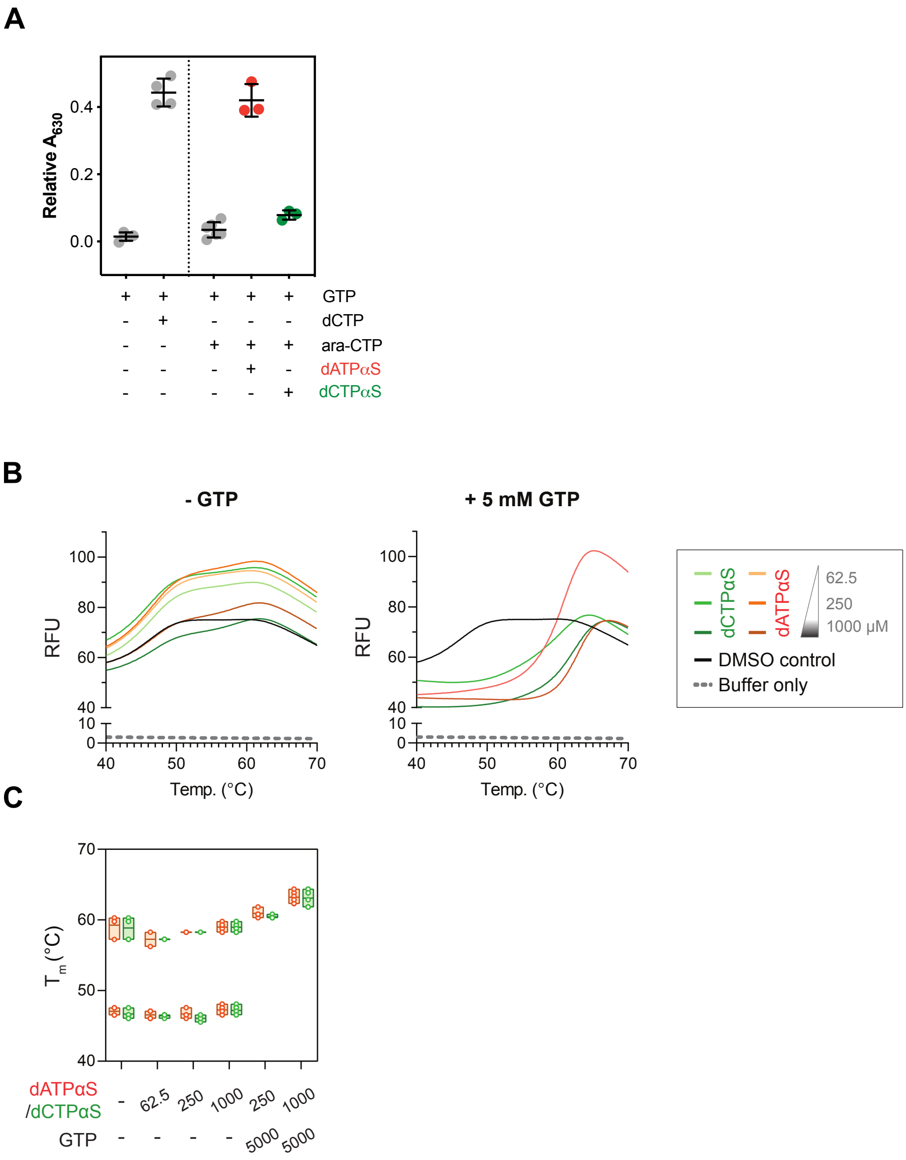
A
Relative A_{630}
0.4
0.2
0.0
GTP
dCTP
ara-CTP
dATPαS
dCTPαS
B
- GTP
+ 5 mM GTP
RFU
Temp. (°C)
dCTPαS
dATPαS
62.5
250
1000 µM
DMSO control
Buffer only
C
T_m (°C)
dATPαS
/dCTPαS
GTP
62.5
250
1000
5000

Figure S13 | Additional biochemical and thermal shift analyses of SAMHD1

A) Recombinant SAMHD1 was incubated with the indicated nucleotide combinations (each 200 µM) in the enzyme-coupled malachite green assay. Absorbance at 630 nm indicates liberated inorganic phosphate. Representative of 2 experiments shown, error bars indicate s.d. of experimental triplicates or quadruplicates.

B) Recombinant SAMHD1 protein (5 µM) was treated with the indicated concentrations of dATPαS or dCTPαS, alone or combined with GTP, for 30 mins before heat-induced denaturation was monitored in the differential scanning fluorimetry (DSF) assay. Mean relative fluorescence units (RFU) of melting curves from 1-2 experiments performed in quadruplicates shown.

C) Melting temperatures of recombinant SAMHD1 proteins as treated in B are summarized. Mean and range are indicated as floating bars with individual replicates plotted from 1-2 experiments.

Supplementary tables

Table S1 | Overview of reported SAMHD1 inhibitors

Inhibitor	Molecule	Mode of action	References
**Vpx**	Protein	Binds to SAMHD1 and recruits it for DCAF1/DDB1 CUL4Aproteasomal degradation machinery for SAMHD1 ubiquitination and degradation	(Hrecka et al, 2011; Laguette et al, 2011)
**SMDU-TP**	Deoxynucleoside triphosphate analogue ((2'S)-2'-methyl-dUTP)	Interferes with SAMHD1 residue Y374; binds to active site but prevents helix A351-V378 conformational change required for dNTP hydrolysis	(Hollenbaugh et al, 2017)
**pppCH2-dU**	dUTP analogue with a methylene bridge connecting the α hosphate and 5′ carbon	Prevents formation of active tetramer and dNTP hydrolysis by antagonistic binding to the allosteric A2 sites	(Seamon et al, 2014)
**Sapacitabine-TP**	Prodrug of 2'-C-cyano-2'-deoxy-1-beta-D-arabinopentofuranosylcytosine (CNDAC)	Predicted inhibitor; (2'S)-2'cyano group which is predicted to block helix A351-V378 conformational change similarly to methyl group in SMDU-TP	(Hollenbaugh et al, 2017)
**DFP-10917**	2'-C-cyano-2'-deoxy-1-beta-D-arabinopentofuranosylcytosine (CNDAC)	Predicted inhibitor; (2'S)-2'cyano group which is predicted to block helix A351-V378 conformational change similarly to methyl group in SMDU-TP	(Hollenbaugh et al, 2017)
**Zn^{2+} salt of cephalosporine C**	Divalent cation	Zn^{2+} salt of the beta-lactam antibiotic cephalosporine C but also Zn^{2+} alone (from chloride salt) was a potent inhibitor of SAMHD1 (IC50 ~2.1μ M); mode of action unknown	(Seamon & Stivers, 2015)
**dGuo**	Deoxyguanosine	Binds guanine nucleoside-specific A1 activator site (IC50 ~488 μM)	(Seamon & Stivers, 2015)
**Aciclovir**	Acycloguanosine	Binds guanine nucleoside-specific A1 activator site (IC50 ~764 μ M)	(Seamon & Stivers, 2015)
**Lomofungin**	Antimicrobial drug	Unknown mechanism. IC50 with dGTP as a substrate 20.1 μM.	(Mauney et al, 2018)

**Troglitazone**	Antidiabetic	Unknown mechanism. IC50 with dGTP as a substrate 23.76 µM.	(Mauney et al, 2018)
**Montelukast**	Leukotriene receptor antagonist	Unknown mechanism. IC50 with dGTP as a substrate 28.62 µM.	(Mauney et al, 2018)
**Pranlukast**	Leukotriene receptor antagonist	Unknown mechanism. IC50 with dGTP as a substrate 33.19 µM.	(Mauney et al, 2018)
**L-thyroxine**	Thyroid hormone	Unknown mechanism. IC50 with dGTP as a substrate 34.17 µM.	(Mauney et al, 2018)
**Ergotamine**	Ergot alklaloid	Unknown mechanism. IC50 with dGTP as a substrate 62.8 µM.	(Mauney et al, 2018)
**Amrinone**	Inhibitor of phosphodiesterase III	Unknown mechanism. IC50 with dGTP as a substrate 66.43 µM.	(Mauney et al, 2018)
**Retinoic Acid**	Vitamin A_1 metabolite	Unknown mechanism. IC50 with dGTP as a substrate 75.67 µM.	(Mauney et al, 2018)
**Ethacrynic Acid**	NKCC2 inhibitor	Unknown mechanism. IC50 with dGTP as a substrate 75.94 µM.	(Mauney et al, 2018)
**Hexestrol**	Synthetic oestrogen	Unknown mechanism. IC50 with dGTP as a substrate 94.66 µM.	(Mauney et al, 2018)
**Tolfenamic acid**	COX inhibitor	Unknown mechanism. IC50 with dGTP as a substrate 178.2 µM.	(Mauney et al, 2018)
**Bexarotene**	Retinoid	Unknown mechanism. IC50 with dGTP as a substrate 216.6 µM.	(Mauney et al, 2018)
**Sulindac**	NSAID	Unknown mechanism. IC50 with dGTP as a substrate 281.7 µM.	(Mauney et al, 2018)
**Zolmitriptan**	Serotonin receptor antagonist	Unknown mechanism. IC50 with dGTP as a substrate 471.2 µM.	(Mauney et al, 2018)
**Nifedipine**	Calcium channel blocker	Unknown mechanism. IC50 with dGTP as a substrate 503.3 µM.	(Mauney et al, 2018)
**Tetracycline**	Inhibitor of bacterial translation	Unknown mechanism. IC50 with dGTP as a substrate 605.7 µM.	(Mauney et al, 2018)

**Nisoldipine**	Calcium channel blocker	Unknown mechanism. IC50 with dGTP as a substrate 783.3 µM.	(Mauney et al, 2018)
**Medroxyprogesterone acetate**	Progestin	Unknown mechanism. IC50 with dGTP as a substrate 1.6 mM.	(Mauney et al, 2018)
**Trifluoperazine**	Antipsychotic	Unknown mechanism. IC50 with dGTP as a substrate 2.4 mM.	(Mauney et al, 2018)
**Primaquine**	Antimalarial drug	Unknown mechanism. IC50 with dGTP as a substrate 4.9 mM.	(Mauney et al, 2018)
**Adapalene**	Retinoid	Unknown mechanism. IC50 with dGTP as a substrate 6.6 mM.	(Mauney et al, 2018)
**Aprepitant**	Neurokinin receptor antagonist	Unknown mechanism. IC50 with dGTP as a substrate 8.1 mM.	(Mauney et al, 2018)
**Tolcapone**	catechol-*O*-methyltransferase inhibitor	Unknown mechanism. IC50 with dGTP as a substrate 8.9 mM.	(Mauney et al, 2018)

Table S2 | Characteristics of AML patients

ID	Age (years)	gender	Complete remission	EFS (days)	OS (days)	risk group	cytogenetics	FLT3	CEBPA.	NPM1	other
adult											
ALG17_010	73	M	N/A	N/A	alive	poor	46,XX	ITD	wt	wt	
ALG17_006	74	M	N/A	N/A	8	favourable	46,XY,inv(16)	N/A	N/A	N/A	*CBFB* rearrangement
ALG17_001	48	M	N/A	N/A	alive	intermediate	47,XY,+8[9]/46,XY[1]	N/A	N/A	N/A	*NOTCH1* mutation
ALG16_037	40	F	N/A	N/A	alive	favourable	46,XX[20]	wt	wt	mut	
ALG16_029	39	M	N/A	N/A	alive	intermediate	47,XY,+8[7]/46,XY[3]	wt	wt	wt	
ALG16_021	71	M	N/A	N/A	489	intermediate	47,XY,+13[2]/46,XX[18]	ITD	wt	mut	
ALG16_018	70	M	N/A	N/A	66	poor	45,XY,-7[19]/46,XY[1]	N/A	N/A	N/A	
ALG16_009	86	M	N/A	N/A	95	favourable	46,XY[20]	wt	wt	mut	
paedaitric											
A318	2	M	Yes	no event	alive	intermediate	46,XY,-19,+mar	N/A	N/A	N/A	t(9;11)
A394	7	M	Yes	no event	alive	intermediate	47,XY,+4[15]/46,XY[10]	wt	wt	wt	
A641	2	F	Yes	no event	alive	intermediate	48,XX,+8,+21c[2]/49,idem,+?22[3]/47,XX,+21c[22]	N/A	N/A	N/A	M. Down
A2202	16	F	Yes	no event	alive	favourable	46,XX,t(15;17)(q24;q21)[11]	N/A	N/A	N/A	APL
A2628	8	M	Yes	1122	alive	intermediate	46,XY,t(7;12)(p1?2;p1?1.2)[4],46,XY,idem,del(5)92,XXYY(q13q31)[2]/46XY[5]	wt	N/A	N/A	
A2953	5	F	Yes	no event	alive	intermediate	46,XX,t(9;11)(p21-22;q23)	ITD	wt	mut	
A3861	7	M	Yes	625	alive	poor	46,XY,t(9;22)(q34;q11)[8]/55,idem,+Y,der(2)t(2;6)(q36;q16),+3,+der(4)t(1;4)(q31;q34),+8,+10,+13,+19,+21,+der(22)t(9;22)[16]	wt	N/A	wt	
A3918	8	F	Yes	no event	alive	intermediate	46,XX,i(7)(p10)[28]/46,XX[3]	wt	wt	mut	
A3922	3	F	Yes	188	206	poor	45,XX,-7[14]/46,XX[2]	wt	wt	mut	
A4003	0.5	F	No	0	3	poor	46XX,t(11;19)(q23;q13.3)	N/A	N/A	N/A	
A4022	1	M	Yes	no event	alive	favourable	46,XY,inv(16)(p13q22)/47,XY,inv(16)(p13q22),+8/46,XY 46,XY,inv(16)(p13;q22)	N/A	N/A	N/A	
A4029	4	F	Yes	no event	alive	intermediate	46,XX[21]	ITD	N/A	mut	

FLT3: fms like tyrosine kinase 3; CEBPA: CCAAT enhancer-binding protein alpha; NPM1: nucleophosmin; CBFB: Core-binding factor subunit beta
APL: acute promyelomonocytic leukaemia; ITD: internal tandem duplication

Table S3 | Summary of pre-clinical studies combining irreversible inhibitors of RNR with ara-C

Cell lines				
**RNRi added to ara-C**	**Cell line(s)**	**Results**	**SAMHD1 status/comment**	**Reference**
HU	HL-60, MOLT-4	3-fold increase of ara-CTP in HL-60, but not in MOLT-4	MOLT-4 cells are SAMHD1-negative whereas HL-60 cells are SAMHD1-positive (Fig. S2C). Consistent with SAMHD1-dependent synergism	(Ahlmann et al, 2001)
HU	CCRF-CEM, HL-60, CHO	Increased ara-CTP in Raji (3.7-fold), HL-60 (3.5-fold) and CHO (8.1-fold), but not in CCRF-CEM	CCRF-CEM cells are SAMHD1-negative whereas HL-60 cells are SAMHD1-positive (Fig. S2C). Consistent with SAMHD1-dependent synergism	(Heinemann et al, 1998)
HU	MOLT-4, HL-60	Increased ara-CTP in HL-60 (2-fold), BALL-1 (2-fold) and Raji (5-fold), not in MOLT-4, CCRF-CEM, U-937; synergism in HL-60, not in MOLT-4	MOLT-4, CEM and U-937 cells are SAMHD1-negative whereas HL-60 cells are SAMHD1-positive ((Laguette et al, 2011) and Fig. S2C). Consistent with SAMHD1-dependent synergism	(Kubota et al, 1988)
HU	CEM, HL-60	Increased ara-CTP in HL-60, not in CEM; 50-fold sensitization to ara-C in HL-60, no sensitization in CEM	CEM cells are SAMHD1-negative whereas HL-60 cells are SAMHD1-positive (Fig. S2C). Consistent with SAMHD1-dependent synergism	(Kubota et al, 1989)
dF-dC	K562	3-fold increase in ara-CTP	K562 cells are SAMHD1-positive (Fig. S2C)	(Gandhi & Plunkett, 1990)

dF-dC	HL-60	3-fold increase of ara-CMP in nuclear DNA; 3-fold increase of apoptosis; 14-fold decrease in clonogenic survival; reduced proportion of cells in S-phase	HL-60 cells are SAMHD1-positive (Fig. S2C)	(Santini et al, 1996)
dF-dC	HL-60	Moderate synergism	HL-60 cells are SAMHD1-positive (Fig. S2C)	(Hubeek et al, 2004)
HU	Ara-C-resistent HL-60	Up to 10-fold reduction in colony formation, no effect on cell cycle distribution and ara-CTP accumulation	HL-60 cells are SAMHD1-positive (Fig. S2C)	(Bhalla et al, 1991)
HU	HL-60	Increase in intracellular ara-CTP, reduced ara-CMP incorporation in nuclear DNA	HL-60 cells are SAMHD1-positive (Fig. S2C)	(Howell et al, 1982)
HU	L1210, HL-60	2-3-fold increase of intracellular ara-CTP, increased ara-CMP incorporation into nuclear DNA, 10-fold decrease in clonogenic survival	HL-60 cells are SAMHD1-positive (Fig. S2C)	(Rauscher & Cadman, 1983)
HU	HL-60, Ara-C-resistent HL-60, U-937	increase in cytotoxicity and apoptosis	U-937 cells are SAMHD1-negative whereas HL-60 cells are SAMHD1-positive ((Laguette et al, 2011) and Fig. S2C)	(Freund et al, 1998)
HU	CCRF-CEM	5-fold increase of ara-CTP, decreased ara-CMP incorporation into DNA, 10-fold reduced colony formation	CCRF-CEM cells are SAMHD1-negative, conflicting results with (Heinemann et al, 1998) (Kubota et al, 1989; Kubota et al, 1988)	(Tanaka et al, 1985)

HU, dF-dC	CCRF-CEM	Increased ara-CMP incorporation into DNA	CCRF-CEM cells are SAMHD1-negative, conflicting results with (Heinemann et al, 1998) (Kubota et al, 1989; Kubota et al, 1988)	(Iwasaki et al, 1997)
HU	Novikoff rat hepatoma cells	4.5-fold increase of ara-C incorporation	unknown	(Plagemann et al, 1978)
3-AP	SW1573, H460	Synergism, 2- to 4-fold increased ara-CTP accumulation, 1.5-fold increased ara-CMP incorporation into DNA	unknown	(Sigmond et al, 2007)
HU	BNML	reduction in proliferation and colony formation; increased ara-CMP in nuclear DNA	unknown	(Colly et al, 1992)
HU	SB	Synergistic, 7.5-fold increase in in ara-C incorporation	unknown	(Streifel & Howell, 1981)
HU	L1210	2.9-fold increase in ara-CTP	unknown	(Walsh et al, 1980)
***Patient blasts* ex vivo**				
**RNRi added to ara-C**	**Cancer type(s)**	**Results**		**Reference**
dF-dC	CML (blast phase)	Strong decrease in clonogenic survival; reduced proportion of cells in S-phase; increased apoptosis		(Santini et al, 1997)
dF-dC, HU	Paediatric AML	synergism		(Hubeek et al, 2005)
dF-dC	CML	Additive effects		(Lech-Maranda et al, 2000)

HU	Paediatric AML, pre-B-ALL, T-ALL, ALL relapse	1.5-fold increase of ara-CTP in AML blasts	(Ahlmann et al, 2001)
HU	AML, ALL	increase of ara-CTP	(Streifel & Howell, 1981)
dF-dC	40 AML samples	No effect on ara-CTP	(Braess et al, 2000)
In vivo studies			
**RNRi added to ara-C**	**Cancer type(s)**	**Results**	**Reference**
dF-dC	L1210 and P388 (mouse)	3-fold and 1.6-fold increase in survival when dF-dC given prior to ara-C	(Lech-Maranda et al, 2000)
HU	L1210 (mouse)	Synergistic increase in survival	(Moran & Straus, 1980)
HU	L1210 (mouse)	2-fold increase in intracellular ara-CTP	(Walsh et al, 1980)

Table S4 | Summary of clinical studies combining irreversible inhibitors of ribonucleotide reductase with ara-C

Regimen	Patients	Results	Reference
100 mg/m^2 ara-C continuous infusion on day 1-5, escalating doses of HU every 6 hours on day 1-5 of a 4 week course	21 patients with relapsed/refractory acute leukaemia or lymphoma	Toxicity: myelosuppression, skin rash<br>Efficacy: complete response in 2 patients (CML (blast crisis and histiocytic lymphoma)	(Howell et al, 1982)
1 g HU every 2-3 h for a total of 10-15 doses prior to 1 mg/kg ara-C every 6 hours for a total of 4 doses (starting 8 h after the last HU dose)	4 patients with AML	Complete remission in 1 patient	(Sauer et al, 1976)
1200 mg/m2 HU 2 h prior to ara-C (dose escalation) on day 1,2,3 and 8,9,10 of 21 day course	26 paediatric patients with ALL, 7 paediatric patients with AML	Toxicity: tolerable<br>Efficacy: complete response in 5 ALL and 1 AML patient, partial response in 3 ALL and 2 AML patients	(Dubowy et al, 2008)
24 h continuous infusion of 3 g HU on day 1 and 4 followed by 200 mg/m^2 ara-C 48 h continuous infusion on day 2 to 3 and 5 to 6	13 patients with AML, 1 patient with CML (blast crisis)	3 complete and 3 partial responses (all AML)	(Zittoun et al, 1985)
500 mg HU every 6 h for 4 doses followed 12 h laters by of 100 mg/m^2 ara-C as continuous infusion on day 1 to 3 and concomitantly 500 mg HU every 4 hours. Cycle duration: 28 days.	21 patients with lymphoma	Toxicity: myelosuppression<br>Efficacy: 4 complete responses, 5 partial responses	(Schilsky et al, 1992; Schilsky et al, 1987)
20 mg/m2 ara-C daily as continuous infusion on day 1 to 21 with 500 mg HU every 12 hours (and 0.25 µg calcitriol every 12 hours)	29 elderly AML patients	Toxicity: myelosuppression<br>Efficacy: 13 complete responses, 10 partial responses	(Slapak et al, 1992)
500 mg HU every 6 hours on day -2 to 0 and every 8 hours from day 0 to 19, 20 mg/m2 ara-C continuous infusion on	19 elderly AML patients	Toxicity: myelosuppression<br>Efficacy: 7 complete responses, 6 partial responses	(Frenette et al, 1995)

day 0 to 19, 250 µg GM-CSF as continuous infusion on day -1 to 6			
50 mg/kg HU on day 1 to 5 of cycle 1, 3 g/m^2 ara-C every 12 hours on day 3 and 4 of cycle 1. In addition asparaginase, etoposide, dexamethasone, cyclophosphamide, mitoxantrone, methotrexate and hydrocortisone during either cycle 1 or 2	6 patients with relapsed/refractory paediatric ALL	Toxicity: myelosuppression, stomatitis<br>Efficacy: 6 complete remission	(Higashigawa et al, 1997)
3-AP 105 mg/m2 as 6 h infusion on day 1 to 5 directly followed by 100 to 800 mg/m2 ara-C as 18 h infusion on day 1 to 5	28 patients with AML, 1 patient with MDS, 3 patients with ALL	Toxicity: mucositis, neuropathy, myelosuppression<br>Efficacy: 4 complete responses (all AML)	(Yee et al, 2006)
3-AP (dose-escalation) from on day 2 to 5 as 2 h infusion 4 h prior to ara-C infusion, ara-C 1 g/m2 on day 1 to 5 as 2 h infusion	25 patients with relapsed/refractory AML	Toxicity: myelosuppression, methaemoglobinaemia<br>Efficacy: 2 complete and 1 partial response	(Odenike et al, 2008)

Supplementary References

Ahlmann M, Lanvers C, Lumkemann K, Rossig C, Freund A, Baumann M, Boos J (2001) Modulation of ara-CTP levels by fludarabine and hydroxyurea in leukemic cells. Leukemia 15**:** 69-73

Bhalla K, Swerdlow P, Grant S (1991) Effects of thymidine and hydroxyurea on the metabolism and cytotoxicity of 1-B-D arabinofuranosylcytosine in highly resistant human leukemia cells. Blood 78**:** 2937-2944

Braess J, Wegendt C, Jahns-Streubel G, Kern W, Keye S, Unterhalt M, Schleyer E, Hiddemann W (2000) Successful modulation of high-dose cytosine arabinoside metabolism in acute myeloid leukaemia by haematopoietic growth factors: no effect of ribonucleotide reductase inhibitors fludarabine and gemcitabine. Br J Haematol 109**:** 388-395

Colly LP, Richel DJ, Arentsen-Honders MW, Kester MG, ter Riet PM, Willemze R (1992) Increase in Ara-C sensitivity in Ara-C sensitive and -resistant leukemia by stimulation of the salvage and inhibition of the de novo pathway. Ann Hematol 65**:** 26-32

Dubowy R, Graham M, Hakami N, Kletzel M, Mahoney D, Newman E, Ravindranath Y, Camitta B (2008) Sequential oral hydroxyurea and intravenous cytosine arabinoside in refractory childhood acute leukemia: a pediatric oncology group phase 1 study. J Pediatr Hematol Oncol 30**:** 353-357

Frenette PS, Desforges JF, Schenkein DP, Rabson A, Slapack CA, Miller KB (1995) Granulocyte-macrophage colony stimulating factor (GM-CSF) priming in the treatment of elderly patients with acute myelogenous leukemia. American journal of hematology 49**:** 48-55

Freund A, Boos J, Harkin S, Schultze-Mosgau M, Veerman G, Peters GJ, Gescher A (1998) Augmentation of 1-beta-D-arabinofuranosylcytosine (Ara-C) cytotoxicity in leukaemia cells by co-administration with antisignalling drugs. European journal of cancer 34**:** 895-901

Gandhi V, Plunkett W (1990) Modulatory activity of 2',2'-difluorodeoxycytidine on the phosphorylation and cytotoxicity of arabinosyl nucleosides. Cancer research 50**:** 3675-3680

Heinemann V, Schulz L, Issels RD, Wilmanns W (1998) Regulation of deoxycytidine kinase by deoxycytidine and deoxycytidine 5' triphosphate in whole leukemia and tumor cells. Adv Exp Med Biol 431**:** 249-253

Higashigawa M, Hori H, Hirayama M, Kawasaki H, Ido M, Azuma E, Sakurai M (1997) Salvage therapy for relapsed or refractory childhood acute lymphocytic leukemia by alternative administration a lymphoid- and myeloid-directed chemotherapeutic regimen consisting of dual modulation of ara-C, hydroxyurea, and etoposide. Leuk Res 21: 811-815

Hollenbaugh JA, Shelton J, Tao S, Amiralaei S, Liu P, Lu X, Goetze RW, Zhou L, Nettles JH, Schinazi RF et al (2017) Substrates and Inhibitors of SAMHD1. PLoS One 12: e0169052

Howell SB, Streifel JA, Pfeifle CE (1982) Modulation of the cellular pharmacology and clinical toxicity of 1-beta-D-arabinofuranosylcytosine. Med Pediatr Oncol 10 Suppl 1: 81-91

Hrecka K, Hao C, Gierszewska M, Swanson SK, Kesik-Brodacka M, Srivastava S, Florens L, Washburn MP, Skowronski J (2011) Vpx relieves inhibition of HIV-1 infection of macrophages mediated by the SAMHD1 protein. Nature 474: 658-661

Hubeek I, Peters GJ, Broekhuizen AJ, Kaspers GJ (2004) Modulation of cytarabine induced cytotoxicity using novel deoxynucleoside analogs in the HL60 cell line. Nucleosides, nucleotides & nucleic acids 23: 1513-1516

Hubeek I, Peters GJ, Broekhuizen AJ, Sargent J, Gibson BE, Creutzig U, Kaspers GJ (2005) Potentiation of in vitro ara-C cytotoxicity by ribonucleotide reductase inhibitors, cyclin-dependent kinase modulators and the DNA repair inhibitor aphidicolin in paediatric acute myeloid leukaemia. Br J Haematol 131: 219-222

Iwasaki H, Huang P, Keating MJ, Plunkett W (1997) Differential incorporation of ara-C, gemcitabine, and fludarabine into replicating and repairing DNA in proliferating human leukemia cells. Blood 90: 270-278

Kubota M, Takimoto T, Kitoh T, Tanizawa A, Akiyama Y, Kiriyama Y, Mikawa H (1989) Ara-CTP metabolism following hydroxyurea or methotrexate treatment in human leukemia cell lines. Adv Exp Med Biol 253b: 363-367

Kubota M, Takimoto T, Tanizawa A, Akiyama Y, Mikawa H (1988) Differential modulation of 1-beta-D-arabinofuranosylcytosine metabolism by hydroxyurea in human leukemic cell lines. Biochem Pharmacol 37: 1745-1749

Laguette N, Sobhian B, Casartelli N, Ringeard M, Chable-Bessia C, Segeral E, Yatim A, Emiliani S, Schwartz O, Benkirane M (2011) SAMHD1 is the dendritic- and myeloid-cell-specific HIV-1 restriction factor counteracted by Vpx. Nature 474: 654-657

Lech-Maranda E, Korycka A, Robak T (2000) The interaction of gemcitabine and cytarabine on murine leukemias L1210 or P388 and on human normal and leukemic cell growth in vitro. Haematologica 85**:** 588-594

Mauney CH, Perrino FW, Hollis T (2018) Identification of Inhibitors of the dNTP Triphosphohydrolase SAMHD1 Using a Novel and Direct High-Throughput Assay. Biochemistry 57**:** 6624-6636

Moran RE, Straus MJ (1980) Synchronization of L1210 leukemia with hydroxyurea infusion and the effect of subsequent pulse dose chemotherapy. Cancer treatment reports 64**:** 81-86

Odenike OM, Larson RA, Gajria D, Dolan ME, Delaney SM, Karrison TG, Ratain MJ, Stock W (2008) Phase I study of the ribonucleotide reductase inhibitor 3-aminopyridine-2-carboxaldehyde-thiosemicarbazone (3-AP) in combination with high dose cytarabine in patients with advanced myeloid leukemia. Investigational new drugs 26**:** 233-239

Plagemann PG, Marz R, Wohlhueter RM (1978) Transport and metabolism of deoxycytidine and 1-beta-D-arabinofuranosylcytosine into cultured Novikoff rat hepatoma cells, relationship to phosphorylation, and regulation of triphosphate synthesis. Cancer research 38**:** 978-989

Rauscher F, 3rd, Cadman E (1983) Biochemical and cytokinetic modulation of L1210 and HL-60 cells by hydroxyurea and effect on 1-beta-D-arabinofuranosylcytosine metabolism and cytotoxicity. Cancer research 43**:** 2688-2693

Santini V, Bernabei A, Gozzini A, Scappini B, Zoccolante A, D'Ippolito G, Figuccia M, Ferrini PR (1997) Apoptotic and antiproliferative effects of gemcitabine and gemcitabine plus Ara-C on blast cells from patients with blast crisis chronic myeloproliferative disorders. Haematologica 82**:** 11-15

Santini V, D'Ippolito G, Bernabei PA, Zoccolante A, Ermini A, Rossi-Ferrini P (1996) Effects of fludarabine and gemcitabine on human acute myeloid leukemia cell line HL 60: direct comparison of cytotoxicity and cellular Ara-C uptake enhancement. Leuk Res 20**:** 37-45

Sauer H, Pelka R, Wilmanns W (1976) [Pharmakokinetics of hydroxy-urea. Therapy of acute myeoblastic leukemias using synchronization and recruitment effects (author' transl)]. Klinische Wochenschrift 54**:** 203-209

Schilsky RL, Ratain MJ, Vokes EE, Vogelzang NJ, Anderson J, Peterson BA (1992) Laboratory and clinical studies of biochemical modulation by hydroxyurea. Seminars in oncology 19**:** 84-89

Schilsky RL, Williams SF, Ultmann JE, Watson S (1987) Sequential hydroxyurea-cytarabine chemotherapy for refractory non-Hodgkin's lymphoma. Journal of clinical oncology : official journal of the American Society of Clinical Oncology 5**:** 419-425

Seamon KJ, Hansen EC, Kadina AP, Kashemirov BA, McKenna CE, Bumpus NN, Stivers JT (2014) Small molecule inhibition of SAMHD1 dNTPase by tetramer destabilization. J Am Chem Soc 136**:** 9822-9825

Seamon KJ, Stivers JT (2015) A High-Throughput Enzyme-Coupled Assay for SAMHD1 dNTPase. J Biomol Screen 20**:** 801-809

Sigmond J, Kamphuis JA, Laan AC, Hoebe EK, Bergman AM, Peters GJ (2007) The synergistic interaction of gemcitabine and cytosine arabinoside with the ribonucleotide reductase inhibitor triapine is schedule dependent. Biochem Pharmacol 73**:** 1548-1557

Slapak CA, Desforges JF, Fogaren T, Miller KB (1992) Treatment of acute myeloid leukemia in the elderly with low-dose cytarabine, hydroxyurea, and calcitriol. American journal of hematology 41**:** 178-183

Streifel JA, Howell SB (1981) Synergistic interaction between 1-beta-D-arabinofuranosylcytosine, thymidine, and hydroxyurea against human B cells and leukemic blasts in vitro. Proc Natl Acad Sci U S A 78**:** 5132-5136

Tanaka M, Kimura K, Yoshida S (1985) Mechanism of synergistic cell killing by hydroxyurea and cytosine arabinoside. Japanese journal of cancer research : Gann 76**:** 729-735

Walsh CT, Craig RW, Agarwal RP (1980) Increased activation of 1-beta-D-arabinofuranosylcytosine by hydroxyurea in L1210 cells. Cancer research 40**:** 3286-3292

Yee KW, Cortes J, Ferrajoli A, Garcia-Manero G, Verstovsek S, Wierda W, Thomas D, Faderl S, King I, O'Brien S M et al (2006) Triapine and cytarabine is an active combination in patients with acute leukemia or myelodysplastic syndrome. Leuk Res 30**:** 813-822

Zhang JH, Chung TD, Oldenburg KR (1999) A Simple Statistical Parameter for Use in Evaluation and Validation of High Throughput Screening Assays. J Biomol Screen 4**:** 67-73

Zittoun R, Marie JP, Zittoun J, Marquet J, Haanen C (1985) Modulation of cytosine arabinoside (ara-C) and high-dose ara-C in acute leukemia. Seminars in oncology 12**:** 139-143
