# Supplementary material for: Ribonucleotide reductase inhibitors suppress SAMHD1 ara‐CTPase activity enhancing cytarabine efficacy
Source: EMBO Mol Med. 2020 Jan 17;12(3):e10419. doi: 10.15252/emmm.201910419 (PMC7059017; doi:10.15252/emmm.201910419)
Supplement: Supplementary file 2 — Expanded View Figures PDF [file EMMM-12-e10419-s002.pdf]

Expanded View Figures

Figure EV1. Phenotypic assay identifies dF-dC as a potential indirect SAMHD1 ara-CTPase inhibitor.

A Dose–response curves for ara-C determined in THP-1 SAMHD1$^{+/+}$ and SAMHD1$^{-/-}$ cells generated to identify sub-lethal concentrations of ara-C. Intersection of vertical dotted lines indicates EC_{10} for the respective cell line. Representative experiment shown of four performed in triplicates.

B Scheme of high-throughput phenotypic screen identifying gemcitabine (dF-dC).

C Proliferation inhibition curves for dF-dC and MK-1775 with and without ara-C at EC_{10} in THP-1 SAMHD1$^{+/+}$ and SAMHD1$^{-/-}$ cells. Representative experiment shown of two performed in triplicates.

D Recombinant SAMHD1 (0.35 μM) was incubated with the indicated compounds (100 μM) prior to addition of dGTP (25 μM) and measurement of dGTPase activity in the enzyme-coupled activity assay. Absorbance at 630 nm, relative to solvent only control, is plotted for each replicate from two independent experiments, mean and SD indicated.

E THP-1 cells were treated with dF-dC (10 μM, 3 h) or DMSO control as indicated prior to cellular thermal shift assay (CETSA) and Western blot analysis. Representative blot and quantification of two independent experiments shown, points normalised to thermostable control NUDT5 band intensity.

© 2020 The Authors

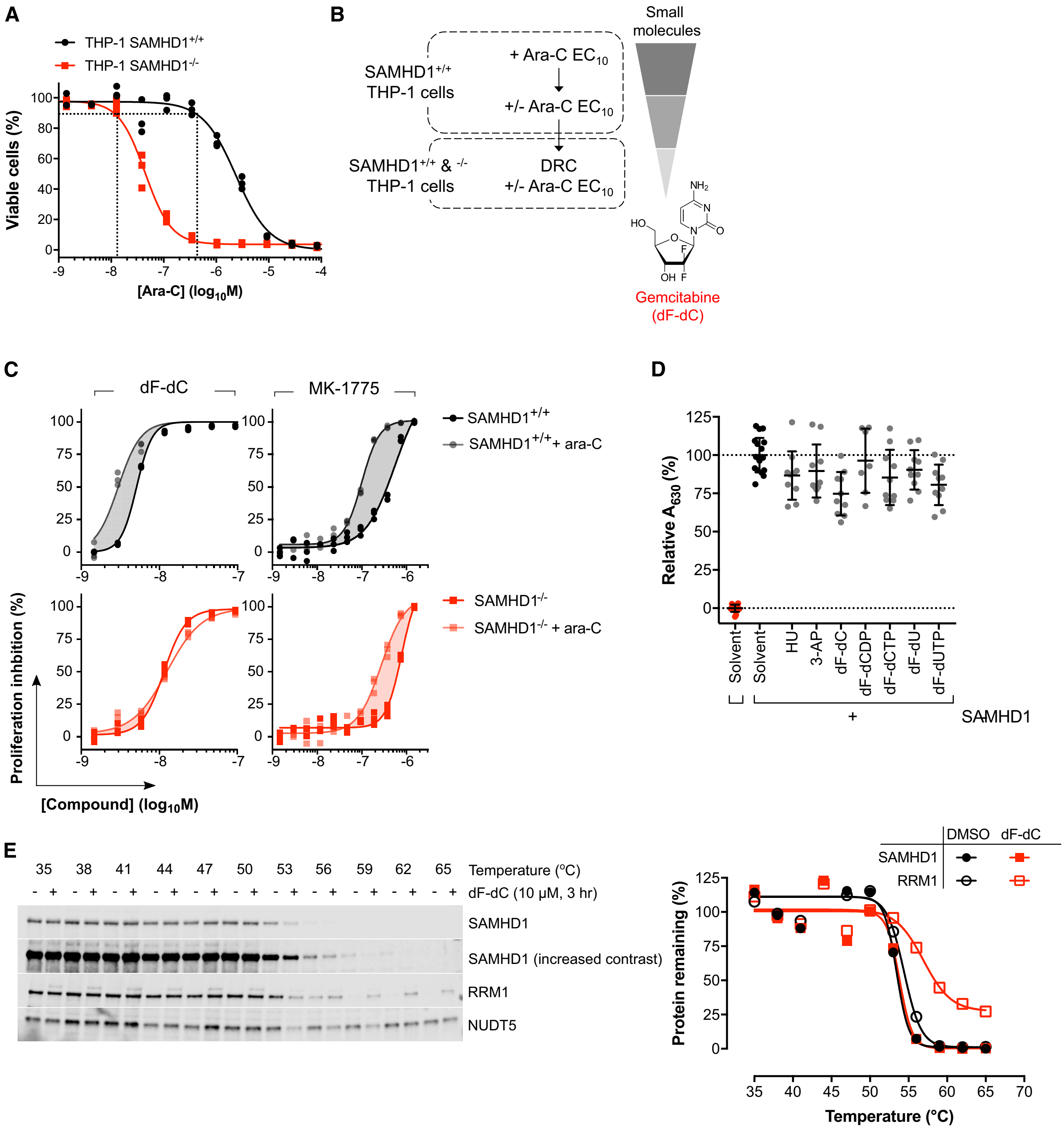

Figure EV1.

 © 2020 The Authors

Figure EV2. RNR inhibitor and ara-C synergy are dependent upon functional SAMHD1 in cancer cell models.

A Proliferation inhibition analysis of ara-C and RNR inhibitor (RNRi) combination treatment in SAMHD1-proficient ($^{+/+}$), SAMHD1-deficient ($^{-/-}$) and rescue (WT, wild-type; H233A, catalytic-dead) cell line pairs. Error bars indicate SEM of two independent experiments for HU and dF-dC, and three independent experiments for 3-AP, each performed in duplicate.

B Drug synergy plots for ara-C and the indicated RNRi in the SAMHD1-proficient ($^{+/+}$), SAMHD1-deficient ($^{-/-}$) and rescue (WT, wild-type; H233A, catalytic-dead) cell line pairs. Zero, > 0 or < 0 corresponds to additive effects, synergy or antagonism, respectively, whilst > 5 indicates strong synergy. Each data point indicates an average excess HSA score from a single dose–response matrix experiment performed in duplicate. The horizontal line and the error bars indicate the mean and SD, respectively; statistical significance was determined using a two-tailed unpaired *t*-test: ns, not significant, $P \geq 0.05$; *$P < 0.05$; **$P < 0.01$; ***$P < 0.001$.

C Immunoblot analysis of lysates prepared from SAMHD1-proficient ($^{+/+}$) or SAMHD1-deficient ($^{-/-}$) THP-1 cells treated for 24 h with ara-C and/or dF-dC, as indicated. Representative of three independent experiments shown.

Data information: Details of statistical testing in (B): For HU: THP-1 $^{+/+}$ vs $^{-/-}$, $n = 7$, $P = 0.0123$, $t = 2.943$, $df = 12$; THP-1 WT vs H233A, $n = 6$, $P = 0.0009$, $t = 4.629$, $df = 10$; HuT-78 $^{+/+}$ vs $^{-/-}$, $n = 6$, $P = 0.0004$, $t = 5.157$, $df = 10$; HL-60 $^{+/+}$ vs $^{-/-}$, $n = 7$, $P = 0.0003$, $t = 5.067$, $df = 12$. For dF-dC: THP-1 $^{+/+}$ vs $^{-/-}$, $n = 6$, $P = 0.0080$, $t = 3.303$, $df = 10$; THP-1 WT vs H233A, $n = 5$, $P = 0.0063$, $t = 3.67$, $df = 8$; HuT-78 $^{+/+}$ vs $^{-/-}$, $n = 5$, $P = 0.0375$, $t = 2.491$, $df = 8$; HL-60 $^{+/+}$ vs $^{-/-}$, $n = 5$, $P = 0.1754$, $t = 1.487$, $df = 8$. For 3-AP: THP-1 $^{+/+}$ vs $^{-/-}$, $n = 4$, $P = 0.0347$, $t = 2.718$, $df = 6$; THP-1 WT vs H233A, $n = 4$, $P = 0.0045$, $t = 4.406$, $df = 6$; HuT-78 $^{+/+}$ vs $^{-/-}$, $n = 4$ and 3, respectively, $P = 0.0010$, $t = 6.824$, $df = 5$; HL-60 $^{+/+}$ vs $^{-/-}$, $n = 4$, $P = 0.2435$, $t = 1.293$, $df = 6$

© 2020 The Authors

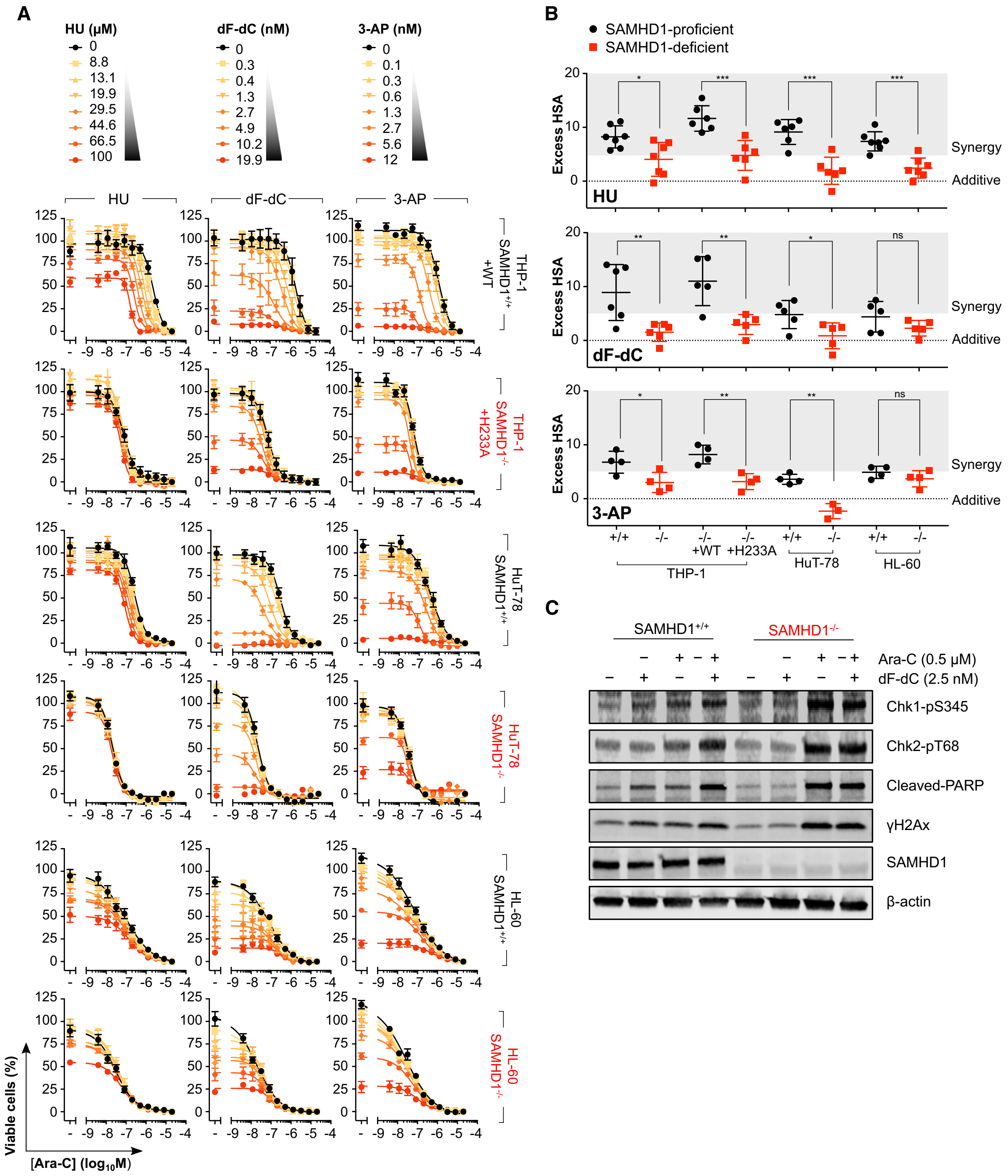

Figure EV2.

© 2020 The Authors

Figure EV3. Allosteric inhibitors of RNR do not synergise with ara-C in a SAMHD1-dependent manner.

A Proliferation inhibition analysis of ara-C and the indicated nucleoside-based, reversible RNR inhibitor (RNRi) combination treatment in SAMHD1-proficient ($^{+/+}$), SAMHD1-deficient ($^{-/-}$) and rescue (WT, wild-type; H233A, catalytic-dead) cell line pairs. Error bars indicate SEM of 2 independent experiments each performed in duplicate. Nucleoside dilution series were as follows: for clofarabine—0.02, 0.03, 0.05, 0.1, 0.18, 0.33 and 0.6 μM; for fludarabine—0.1, 0.3, 0.8, 2, 5.1, 12.8 and 32.7 μM; for cladribine—0.01, 0.02, 0.04, 0.09, 0.17, 0.34 and 0.66 μM.

B Half-maximal effective concentration (EC_{50}) values for ara-C plotted as a function of nucleoside-based, reversible RNRi concentration in SAMHD1-proficient ($^{+/+}$), SAMHD1-deficient ($^{-/-}$) and rescue (WT, wild-type; H233A, catalytic-dead) THP-1 cell line pairs. Ara-C EC_{50} values for SAMHD1$^{+/+}$ and SAMHD1$^{-/-}$ THP-1 cells in the absence of nucleoside-based, reversible RNRi are indicated by the black and red dotted line, respectively. Error bars indicate SEM of two independent experiments each performed in duplicate.

C Drug synergy plots for ara-C and the indicated nucleoside-based, reversible RNRi in the SAMHD1-proficient ($^{+/+}$), SAMHD1-deficient ($^{-/-}$) and rescue (WT, wild-type; H233A, catalytic-dead) cell line pairs. Zero, > 0 or < 0 corresponds to additive effects, synergy or antagonism, respectively, whilst > 5 indicates strong synergy. Each data point indicates an average delta score from a single dose–response matrix experiment performed in duplicate. The horizontal line and the error bars indicate the mean and SD, respectively; statistical significance was determined using a two-tailed unpaired *t*-test: ns, not significant, $P \geq 0.05$; *$P < 0.05$.

D Drug synergy plots for ara-C and the indicated nucleoside-based, reversible RNRi in the SAMHD1-proficient ($^{+/+}$), SAMHD1-deficient ($^{-/-}$) and rescue (WT, wild-type; H233A, catalytic-dead) cell line pairs. Zero, > 0 or < 0 corresponds to additive effects, synergy or antagonism, respectively, whilst > 5 indicates strong synergy. Each data point indicates an average excess HSA score from a single dose–response matrix experiment performed in duplicate. The horizontal line and the error bars indicate the mean and SD, respectively; statistical significance was determined using a two-tailed unpaired *t*-test: ns, not significant, $P \geq 0.05$; ***$P < 0.001$.

Data information: Details of statistical testing in (C): For clofarabine: THP-1 $^{+/+}$ vs $^{-/-}$, $n = 3$, $P = 0.8356$, $t = 0.2215$, $df = 4$; THP-1 WT vs H233A, $n = 3$, $P = 0.1237$, $t = 1.945$, $df = 4$; HuT-78 $^{+/+}$ vs $^{-/-}$, $n = 3$, $P = 0.0126$, $t = 4.304$, $df = 4$; HL-60 $^{+/+}$ vs $^{-/-}$, $n = 3$, $P = 0.4523$, $t = 0.8317$, $df = 4$. For fludarabine: THP-1 $^{+/+}$ vs $^{-/-}$, $n = 3$, $P = 0.1406$, $t = 1.834$, $df = 4$; THP-1 WT vs H233A, $n = 3$, $P = 0.0988$, $t = 2.143$, $df = 4$; HuT-78 $^{+/+}$ vs $^{-/-}$, $n = 3$, $P = 0.5989$, $t = 0.5704$, $df = 4$; HL-60 $^{+/+}$ vs $^{-/-}$, $n = 3$, $P = 0.0341$, $t = 3.162$, $df = 4$. For cladribine: THP-1 $^{+/+}$ vs $^{-/-}$, $n = 3$, $P = 0.7389$, $t = 0.3579$, $df = 4$; THP-1 WT vs H233A, $n = 3$, $P = 0.4185$, $t = 0.901$, $df = 4$; HuT-78 $^{+/+}$ vs $^{-/-}$, $n = 3$, $P = 0.8805$, $t = 0.1601$, $df = 4$; HL-60 $^{+/+}$ vs $^{-/-}$, $n = 3$, $P = 0.0204$, $t = 3.724$, $df = 4$. Details of statistical testing in (D): for clofarabine: THP-1 $^{+/+}$ vs $^{-/-}$, $n = 3$, $P = 0.2689$, $t = 1.283$, $df = 4$; THP-1 WT vs H233A, $n = 3$, $P = 0.0740$, $t = 2.405$, $df = 4$; HuT-78 $^{+/+}$ vs $^{-/-}$, $n = 3$, $P = 0.9855$, $t = 0.01939$, $df = 4$; HL-60 $^{+/+}$ vs $^{-/-}$, $n = 3$, $P = 0.3830$, $t = 0.9789$, $df = 4$. For fludarabine: THP-1 $^{+/+}$ vs $^{-/-}$, $n = 3$, $P = 0.5879$, $t = 0.5884$, $df = 4$; THP-1 WT vs H233A, $n = 3$, $P = 0.6425$, $t = 0.5012$, $df = 4$; HuT-78 $^{+/+}$ vs $^{-/-}$, $n = 3$, $P = 0.3070$, $t = 1.17$, $df = 4$; HL-60 $^{+/+}$ vs $^{-/-}$, $n = 3$, $P = 0.1353$, $t = 1.867$, $df = 4$. For cladribine: THP-1 $^{+/+}$ vs $^{-/-}$, $n = 3$, $P = 0.3265$, $t = 1.117$, $df = 4$; THP-1 WT vs H233A, $n = 3$, $P = 0.8537$, $t = 0.1966$, $df = 4$; HuT-78 $^{+/+}$ vs $^{-/-}$, $n = 3$, $P = 0.7201$, $t = 0.3846$, $df = 4$; HL-60 $^{+/+}$ vs $^{-/-}$, $n = 3$, $P = 0.0004$, $t = 10.63$, $df = 4$.

© 2020 The Authors

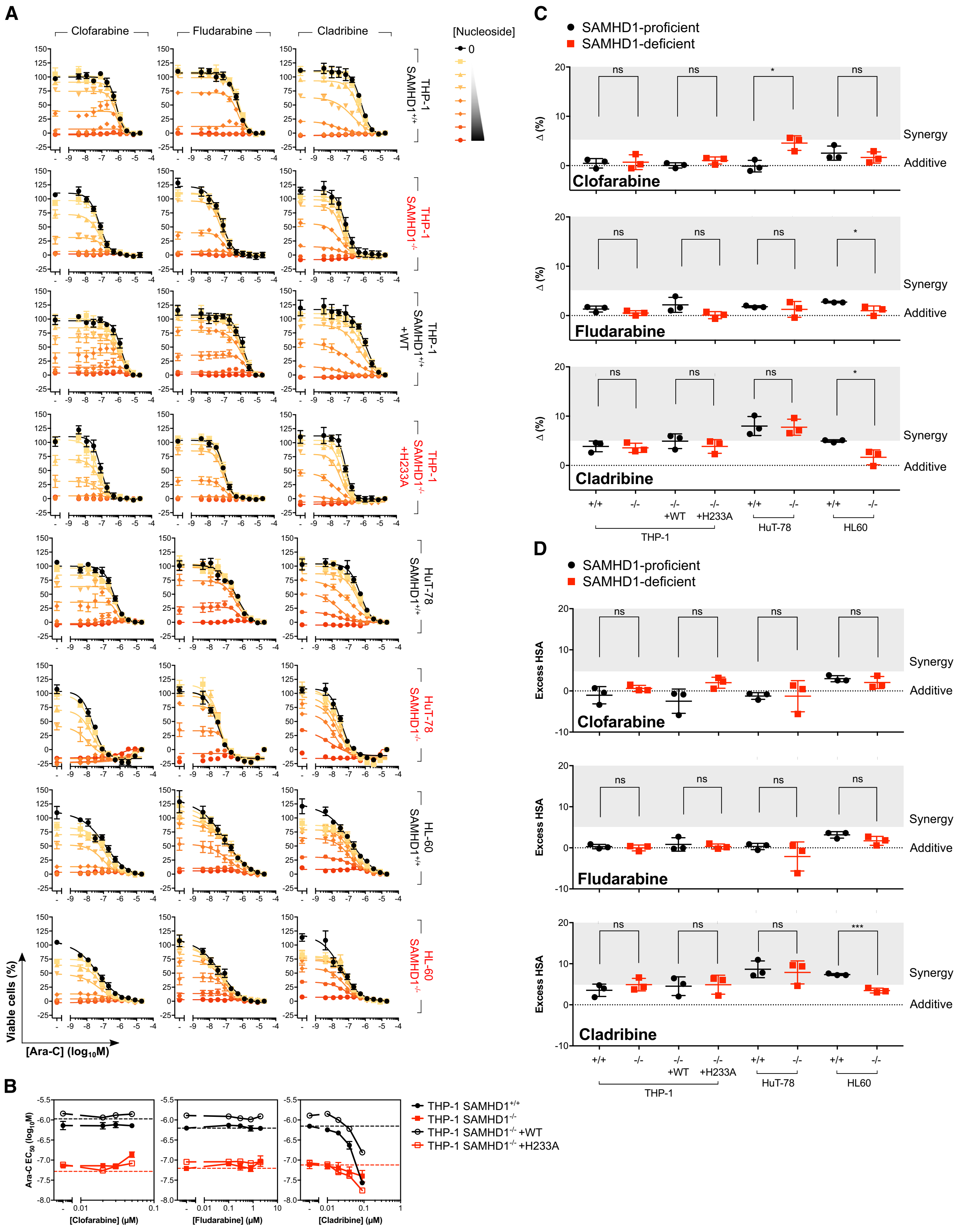

Figure EV3.

© 2020 The Authors

Figure EV4. Assessment of haematological toxicity of ara-C and HU an immunocompetent murine AML model.

A–F Twenty-four hours after a 5-day course of treatment with vehicle (PBS, black), HU (grey), ara-C (orange) or ara-C plus HU (red), peripheral blood was taken, and total white blood cell count (WBC, A), red blood cell count (RBC, B), haemoglobin concentration (C), mean corpuscular volume (MCV, D), mean corpuscular haemoglobin (MCH, E) and platelet count (F) were determined using a haematology analyser.

G At sacrifice, bone marrow cellularity was determined following aspiration from one femur per animal using a haematology analyser.

H At sacrifice, spleen weight was measured following necropsy.

Data information: Statistical significance was determined using a two-tailed unpaired t-test: *$P < 0.05$; **$P < 0.01$; ***$P < 0.001$. Details of the statistical tests: A: vehicle vs HU, $n = 5$ and 4, respectively, $P = 0.2780$, $t = 1.176$, $df = 7$; vehicle vs ara-C, $n = 5$ and 4, respectively, $P = 0.0010$, $t = 5.406$, $df = 7$; vehicle vs ara-C + HU, $n = 5$ and 4, respectively, $P = 0.0002$, $t = 7.250$, $df = 7$; HU vs ara-C, $n = 5$ and 5, respectively, $P = 0.0086$, $t = 3.461$, $df = 8$; HU vs ara-C + HU, $n = 5$ and 5, respectively, $P = 0.0021$, $t = 4.447$, $df = 8$; ara-C vs ara-C + HU, $n = 5$ and 5, respectively, $P = 0.3570$, $t = 0.9775$, $df = 8$. B: vehicle vs HU, $n = 5$ and 4, respectively, $P = 0.0483$, $t = 2.389$, $df = 7$; vehicle vs ara-C, $n = 5$ and 4, respectively, $P = 0.0079$, $t = 3.672$, $df = 7$; vehicle vs ara-C + HU, $n = 5$ and 4, respectively, $P = 0.0296$, $t = 2.724$, $df = 7$; HU vs ara-C, $n = 5$ and 5, respectively, $P = 0.5833$, $t = 0.5761$, $df = 8$; HU vs ara-C + HU, $n = 5$ and 5, respectively, $P = 0.2421$, $t = 1.263$, $df = 8$; ara-C vs ara-C + HU, $n = 5$ and 5, respectively, $P = 0.3313$, $t = 1.034$, $df = 8$. C: vehicle vs HU, $n = 5$ and 4, respectively, $P = 0.0356$, $t = 2.597$, $df = 7$; vehicle vs ara-C, $n = 5$ and 4, respectively, $P = 0.0017$, $t = 4.944$, $df = 7$; vehicle vs ara-C + HU, $n = 5$ and 4, respectively, $P = 0.0079$, $t = 3.680$, $df = 7$; HU vs ara-C, $n = 5$ and 5, respectively, $P = 0.6525$, $t = 0.4677$, $df = 8$; HU vs ara-C + HU, $n = 5$ and 5, respectively, $P = 0.1551$, $t = 1.570$, $df = 8$; ara-C vs ara-C + HU, $n = 5$ and 5, respectively, $P = 0.1675$, $t = 1.518$, $df = 8$. D: vehicle vs HU, $n = 5$ and 4, respectively, $P = 0.5562$, $t = 0.6180$, $df = 7$; vehicle vs ara-C, $n = 5$ and 4, respectively, $P = 0.0226$, $t = 2.910$, $df = 7$; vehicle vs ara-C + HU, $n = 5$ and 4, respectively, $P = 0.0213$, $t = 2.953$, $df = 7$; HU vs ara-C, $n = 5$ and 5, respectively, $P = 0.3678$, $t = 0.9544$, $df = 8$; HU vs ara-C + HU, $n = 5$ and 5, respectively, $P = 0.2705$, $t = 1.184$, $df = 8$; ara-C vs ara-C + HU, $n = 5$ and 5, respectively, $P = 0.6115$, $t = 0.5285$, $df = 8$. E: vehicle vs HU, $n = 5$ and 4, respectively, $P = 0.9723$, $t = 0.03957$, $df = 7$; vehicle vs ara-C, $n = 5$ and 4, respectively, $P = 0.7872$, $t = 0.2804$, $df = 7$; vehicle vs ara-C + HU, $n = 5$ and 4, respectively, $P = 0.8842$, $t = 0.1511$, $df = 7$; HU vs ara-C, $n = 5$ and 5, respectively, $P = 0.7834$, $t = 0.2843$, $df = 8$; HU vs ara-C + HU, $n = 5$ and 5, respectively, $P = 0.8866$, $t = 0.1473$, $df = 8$; ara-C vs ara-C + HU, $n = 5$ and 5, respectively, $P = 0.9641$, $t = 0.04637$, $df = 8$. F: vehicle vs HU, $n = 5$ and 4, respectively, $P = 0.9391$, $t = 0.07915$, $df = 7$; vehicle vs ara-C, $n = 5$ and 4, respectively, $P = 0.7976$, $t = 0.2664$, $df = 7$; vehicle vs ara-C + HU, $n = 5$ and 4, respectively, $P = 0.7873$, $t = 0.2804$, $df = 7$; HU vs ara-C, $n = 5$ and 5, respectively, $P = 0.9195$, $t = 0.1043$, $df = 8$; HU vs ara-C + HU, $n = 5$ and 5, respectively, $P = 0.7802$, $t = 0.2887$, $df = 8$; ara-C vs ara-C + HU, $n = 5$ and 5, respectively, $P = 0.3998$, $t = 0.8894$, $df = 8$. G: vehicle vs HU, $n = 4$ and 4, respectively, $P = 0.4684$, $t = 0.7739$, $df = 6$; vehicle vs ara-C, $n = 4$ and 5, respectively, $P = 0.8968$, $t = 0.1345$, $df = 7$; vehicle vs ara-C + HU, $n = 4$ and 5, respectively, $P = 0.2041$, $t = 1.401$, $df = 7$; HU vs ara-C, $n = 4$ and 5, respectively, $P = 0.3305$, $t = 1.046$, $df = 7$; HU vs ara-C + HU, $n = 4$ and 5, respectively, $P = 0.4814$, $t = 0.8597$, $df = 7$; ara-C vs ara-C + HU, $n = 5$ and 5, respectively, $P = 0.0998$, $t = 1.867$, $df = 8$. H: vehicle vs HU, $n = 4$ and 4, respectively, $P = 0.2715$, $t = 0.1211$, $df = 6$; vehicle vs ara-C, $n = 4$ and 5, respectively, $P = 0.3463$, $t = 1.010$, $df = 7$; vehicle vs ara-C + HU, $n = 4$ and 5, respectively, $P = 0.1324$, $t = 1.703$, $df = 7$; HU vs ara-C, $n = 4$ and 5, respectively, $P = 0.8783$, $t = 0.1589$, $df = 7$; HU vs ara-C + HU, $n = 4$ and 5, respectively, $P = 0.7940$, $t = 0.2713$, $df = 7$; ara-C vs ara-C + HU, $n = 5$ and 5, respectively, $P = 0.9897$, $t = 0.1326$, $df = 8$.

© 2020 The Authors

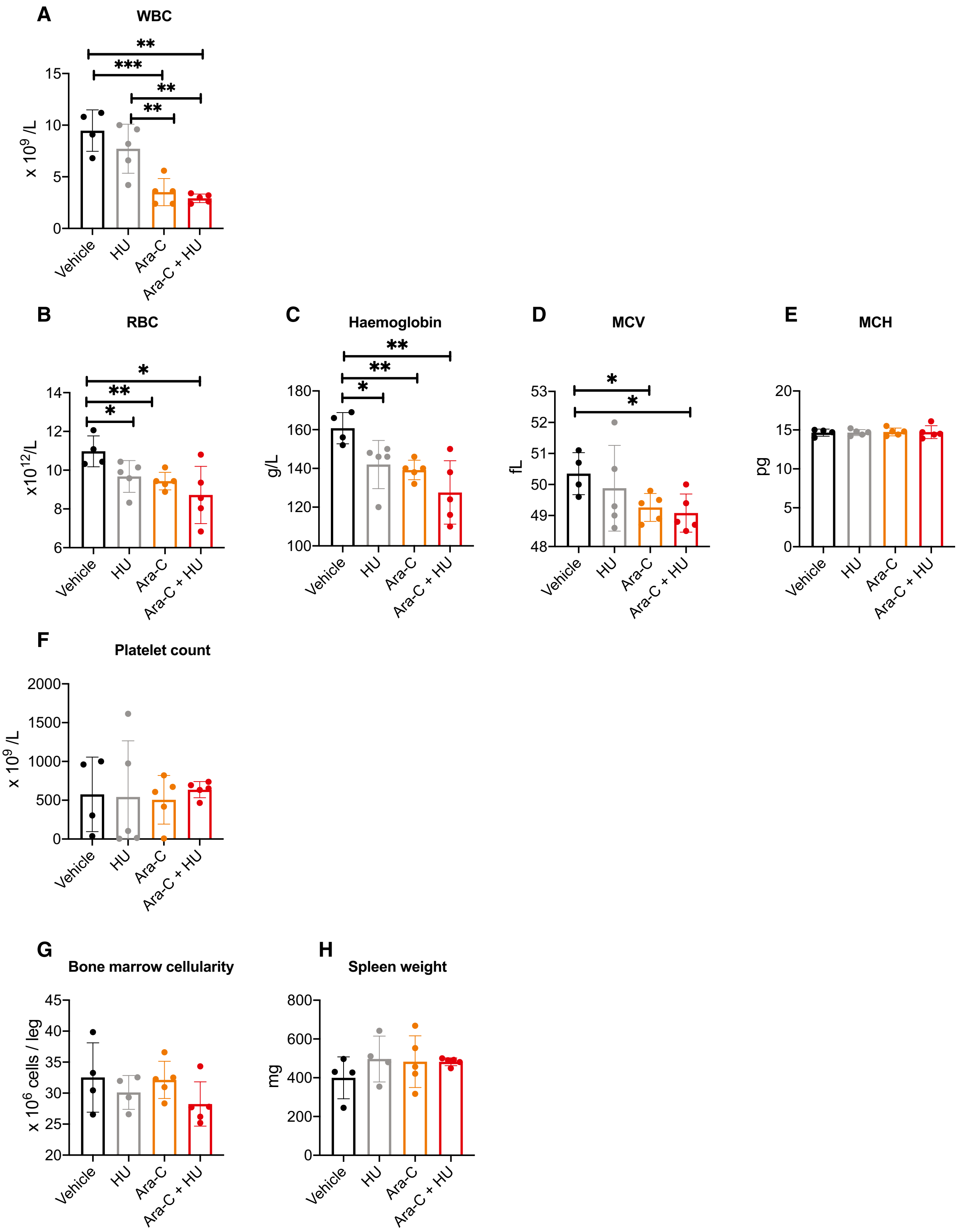

Figure EV4.

© 2020 The Authors

Figure EV5. RNRi-treated cells retain tetrameric SAMHD1.

A SAMHD1-proficient ($^{+/+}$) or SAMHD1-deficient ($^{-/-}$) THP-1 cells were treated with chemical cross-linker disuccinimidyl glutarate (DSG; 5, 2.5, 1.25, 0.6 and 0.3 mM) prior to Western blot analysis. Oligomeric SAMHD1 species are indicated, blot representative of two independent experiments shown.

B SAMHD1-proficient ($^{+/+}$) or SAMHD1-deficient ($^{-/-}$) THP-1 cells, the latter with rescue expression of dimerisation-dead mutant Y146S/Y154S, were subjected to chemical cross-linking and Western blot analysis. Oligomeric SAMHD1 species are indicated, blot representative of two independent experiments.

C THP-1 cells were treated with the indicated dose of HU for 24 h before chemical cross-linking and Western blot analysis. Oligomeric SAMHD1 species are indicated, blot representative of three independent experiments shown.

D THP-1 cells were treated with the indicated dose of dF-dC for 24 h before chemical cross-linking and Western blot analysis. Oligomeric SAMHD1 species are indicated, blot representative of three independent experiments shown.

E THP-1 cells were treated with HU (50 μM, 24 h) or solvent control as indicated prior to cellular thermal shift assay (CETSA) and Western blot analysis. Representative blot and quantification of three independent experiments shown, points normalised to thermostable control SOD-1 band intensity.

F THP-1 cells were treated with dF-dC (2.5 nM, 24 h) or DMSO control as indicated prior to cellular thermal shift assay (CETSA) and Western blot analysis. Representative blot and quantification of two independent experiments shown, points normalised to thermostable control SOD-1 band intensity.

G THP-1 cells expressing endogenous SAMHD1 (WT) or SAMHD1-deficient THP-1 cells with rescue expression of dimerisation-dead mutant Y146S/Y154S (YY) were subjected to CETSA and Western blot analysis. Representative blot and quantification of two independent experiments shown, points normalised to thermostable control SOD-1 band intensity.

H Model of selective inhibition of SAMHD1 ara-CTPase by RNR inhibitors. Inhibition of RNR reduces supply of *de novo* dNTPs, resulting in net depletion of purine dNTPs (in particular dATP), whilst activating dCK, resulting in net elevation of pyrimidine dNTPs (in particular dCTP). Effectively, the preferred occupation of the second allosteric site of SAMHD1 will be shifted from dATP to dCTP, leading to selective loss of the ara-CTPase activity of SAMHD1.

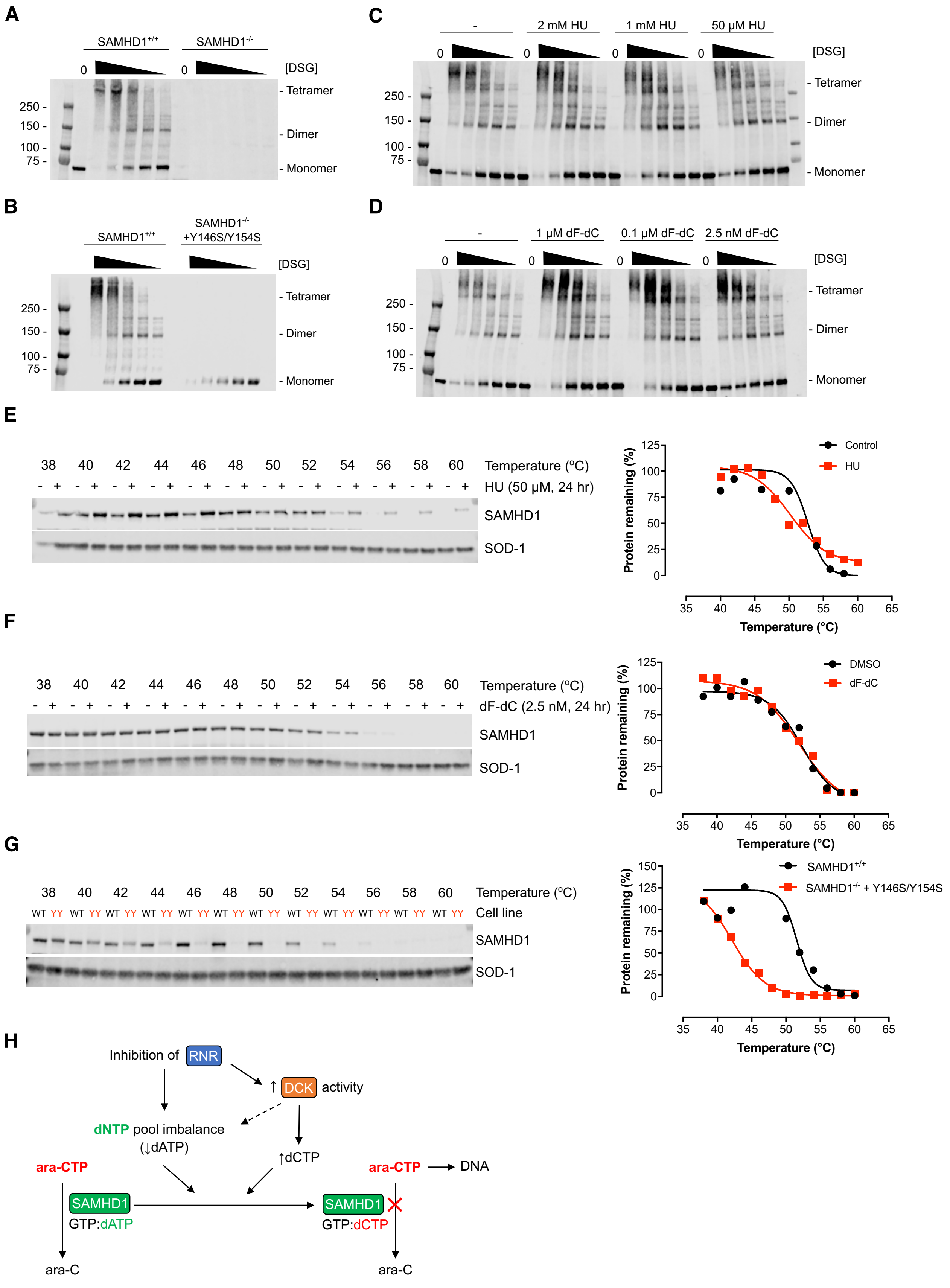

Figure EV5.

 © 2020 The Authors
