# Supplementary figures and images for: Ribonucleotide reductase inhibitors suppress SAMHD1 ara‐CTPase activity enhancing cytarabine efficacy
Source: EMBO Mol Med. 2020 Jan 17;12(3):e10419. doi: 10.15252/emmm.201910419 (PMC7059017; doi:10.15252/emmm.201910419)

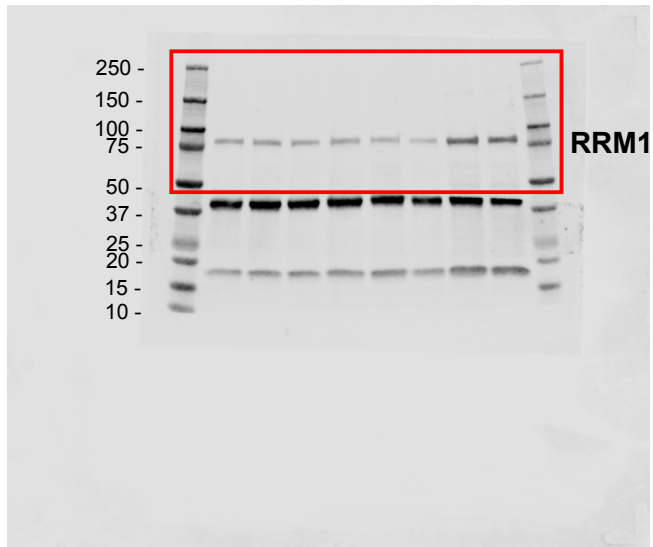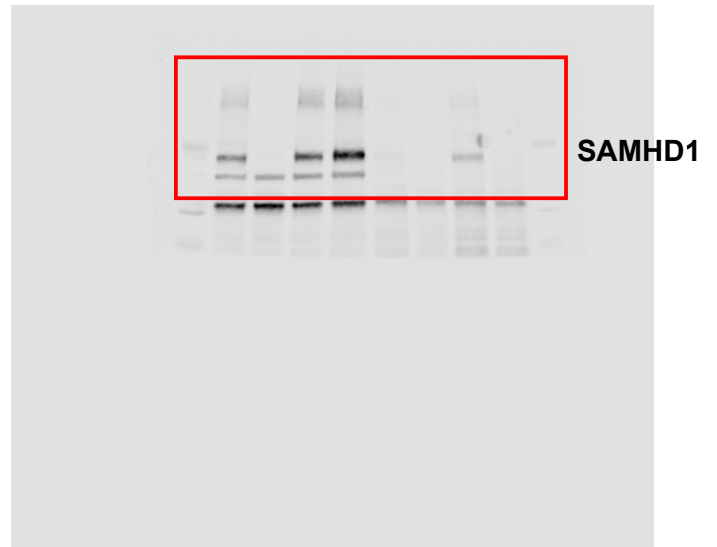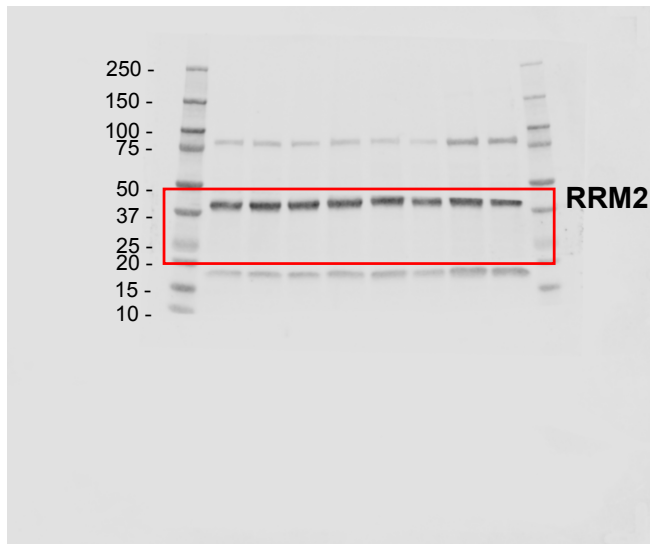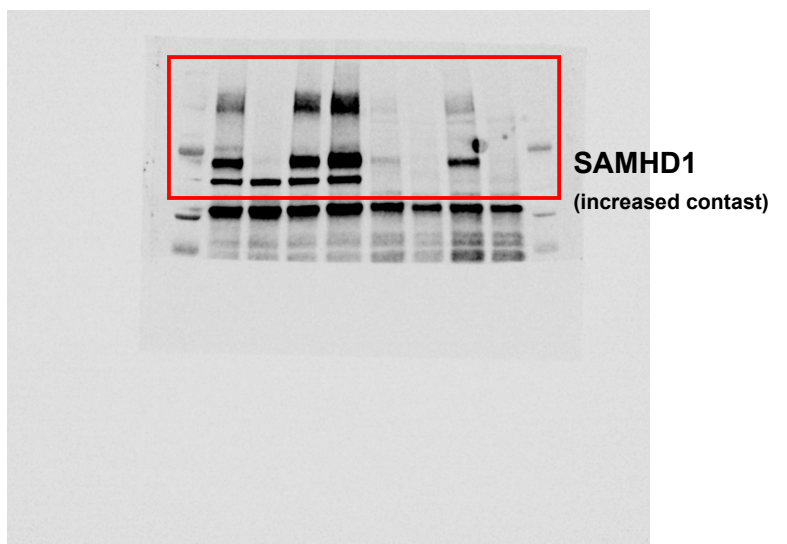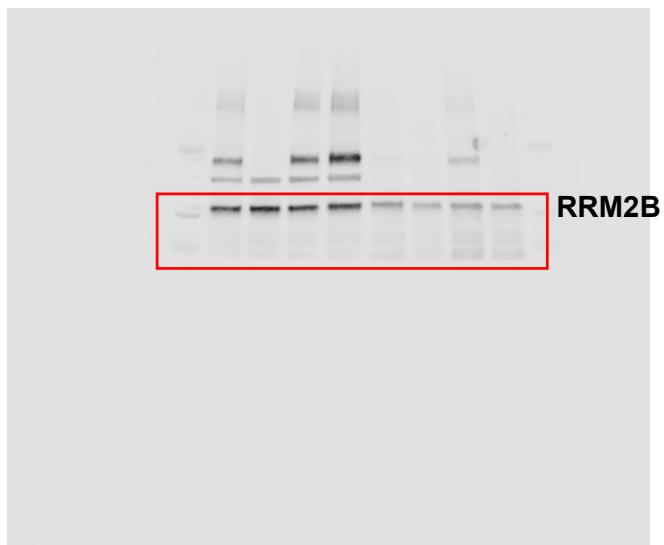

Supplement: Supplementary file 4 — Source Data for Figure 1 [file EMMM-12-e10419-s003.zip › emmm201910419-sup-0003-SDataFig1B.pdf]

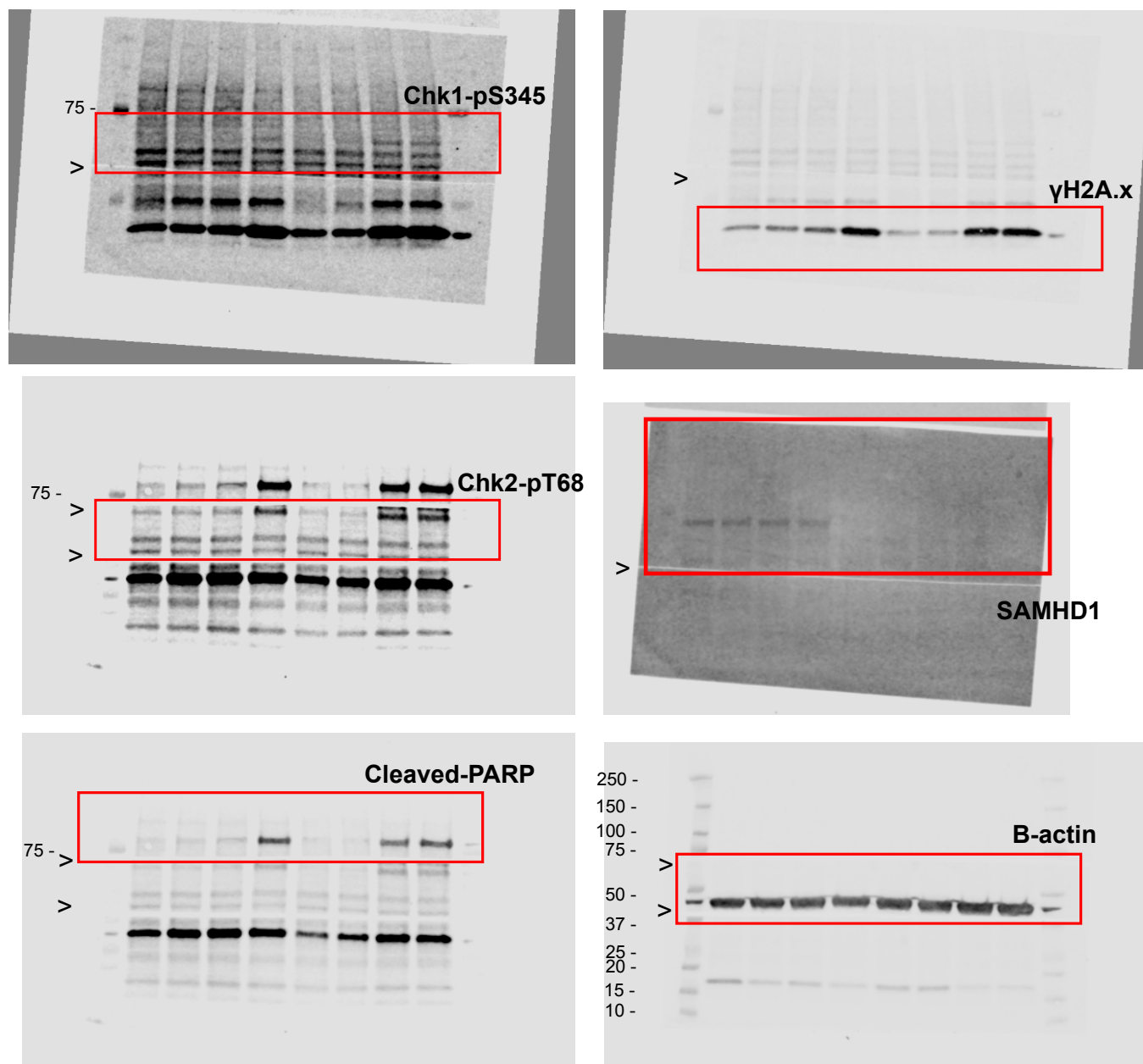

> indicates membrane cut site

Supplement: Supplementary file 4 — Source Data for Figure 1 [file EMMM-12-e10419-s003.zip › emmm201910419-sup-0004-SDataFigG.pdf]

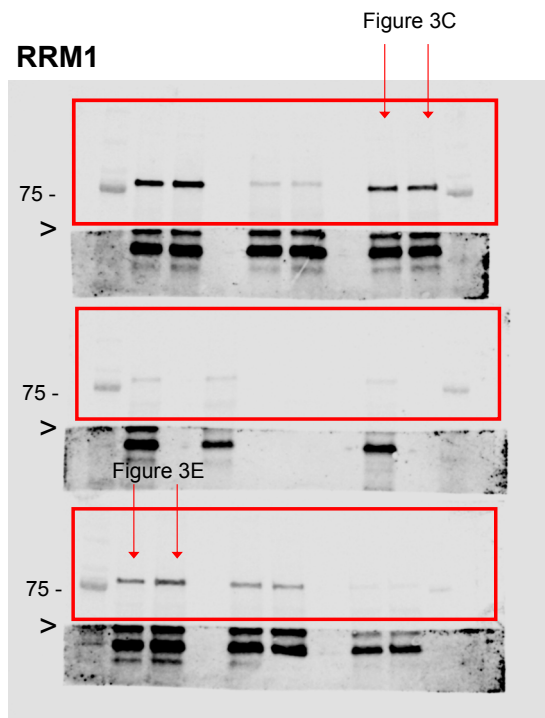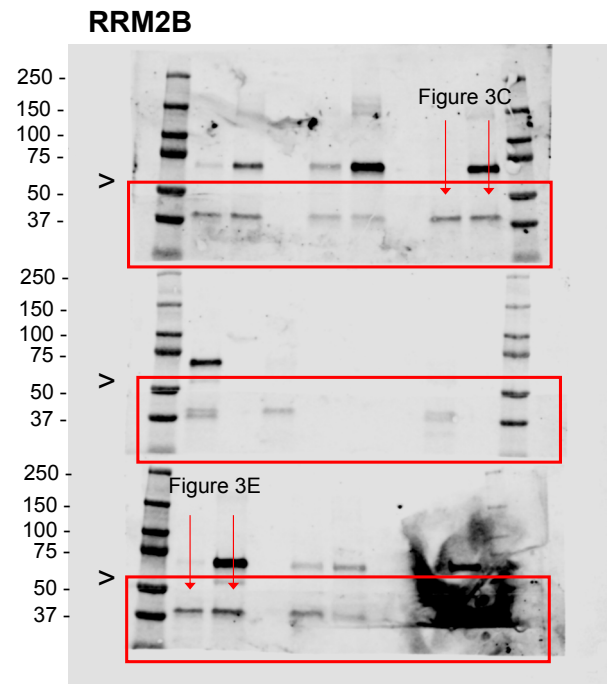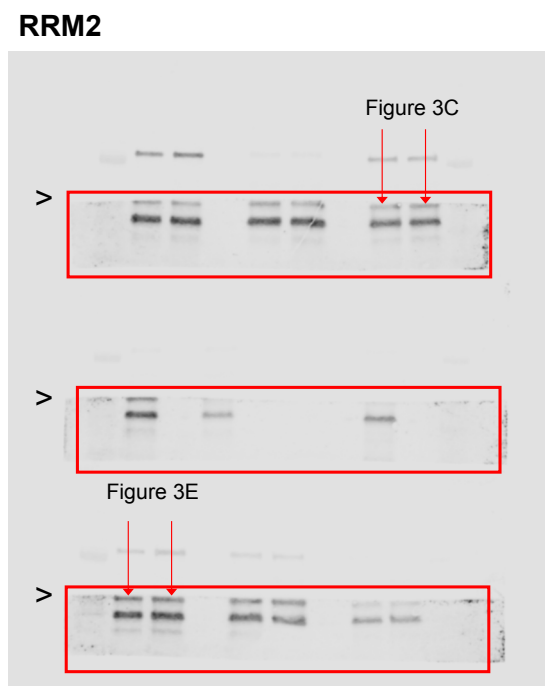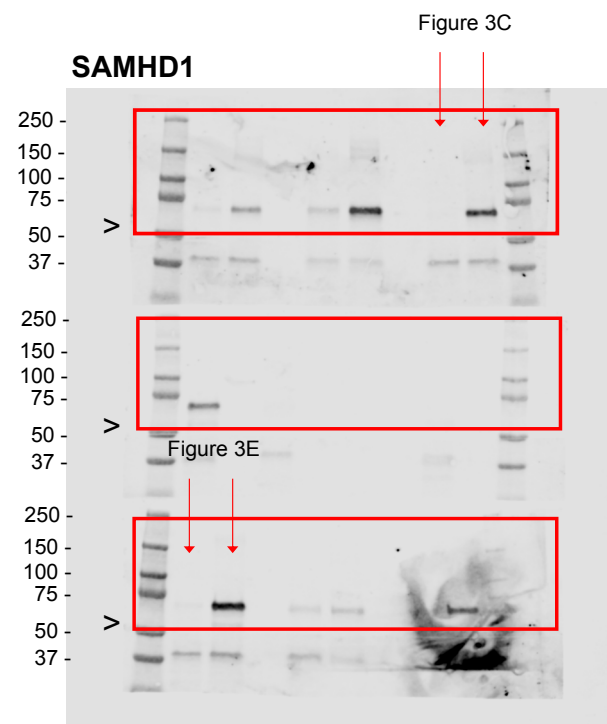

> indicates membrane cut site

Supplement: Supplementary file 6 — Source Data for Figure 3 [file EMMM-12-e10419-s005.pdf]
